# Supplementary material for: Perylenetetracarboxylic acid nanosheets with internal electric fields and anisotropic charge migration for photocatalytic hydrogen evolution
Source: Nat Commun. 2022 Apr 19;13:2067. doi: 10.1038/s41467-022-29826-z (PMC9018690; doi:10.1038/s41467-022-29826-z)
Supplement: Supplementary file 1 — Supplementary Information [file 41467_2022_29826_MOESM1_ESM.pdf]

Supplementary Information

Perylenetetracarboxylic Acid Nanosheets with Internal Electric Fields and Anisotropic Charge Migration for Photocatalytic Hydrogen Evolution

Yan Guo[1,2], Qixin Zhou[2], Jun Nan[1], Wenxin Shi[3], Fuyi Cui[3] and Yongfa Zhu[2]*

[1]School of Environment, Harbin Institute of Technology, Harbin, 150090, China

[2]Department of Chemistry, Tsinghua University, Beijing, 100084, China

[3]College of Environment and Ecology, Chongqing University, Chongqing, 400044, China

*E-mails: Zhuyf@tsinghua.edu.cn

This PDF file includes:

Supplementary Figures 1 to 45

Supplementary Notes 1 to 8

Supplementary Methods 1 to 3

Supplementary Tables 1 to 4

Supplementary References

Table of contents

1. Supplementary Figures. 7

Supplementary Figure 1. Chemical structure characterization of PTA. (a) Solid 13CNMR spectrum, (b) FT-IR and (c) TOF-SIMS of PTA. 7

Supplementary Figure 2. X-ray photoelectron spectroscopy (XPS) analysis of PTA. (a) C 1*s* and (b) O 1*s*. 9

Supplementary Figure 3. Chemical structure characterization of PTA precursor PTCDA. (a) Solid ^{13}CNMR spectrum, (b) FT-IR and (c) TOF-SIMS of PTCDA. 10

Supplementary Figure 4. Elemental analysis of two selections. TEM image with mapping data of PTA nanosheets. 11

Supplementary Figure 5. Optimized PTA cell. The front view (left) and side view (right) of PTA cell. 12

Supplementary Figure 6. The morphology and crystal structure of PTA precursor PTCDA were characterized. (a) XRD, (b) TEM, (c) AFM and (d) its height value of PTCDA. 13

Supplementary Figure 7. Morphological characteristics of PTA nanosheets. (a) FESEM and (b) effective surface area determination (BET measurements) of PTA. 14

Supplementary Figure 8. Effect of water vapor on the height of PTA nanosheets. PTA nanosheets height at (a) dry-state (25% humidity) and (b) wet-state (Process in 90% humidity environment for 30min). Test conditions: Using AFM equipped with environmental control scanner, and the ambient humidity is controlled by adding water vapor in the humidity cell. 15

Supplementary Figure 9. Effect of water vapor on the morphology of PTA nanosheets. The SEM of (a) Dry-state and (b) wet-state of PTA (H_2O: 33000 ppm). Scan the water-absorbent sample by lowering the test vacuum (Acceleration voltage: 15kV & 2 kV). . 16

Supplementary Figure 10. Effect of water environment on the structure of PTA nanosheets. (a) UV-vis of PTA in alkali and water, respectively. (b) FT-IR of PTA adsorb water. 17

Supplementary Figure 11. Effect of water environment on the thickness of PTA nanosheets. Small Angle X-ray Scattering (SAXS) data for PTA in water (1mg mL^{-1}). (The data is fitted using lamellar model.) 18

Supplementary Figure 12. Effect of water environment on the crystal structure of PTA nanosheets. X-rays diffraction (XRD) of (a) PTA sample and (b) external standard Si powder. 19

Supplementary Figure 13. Optimized PTA crystals in a water environment. Optimized model by PM6 semi-empirical method (Black: carbon, yellow: oxygen, white: hydrogen) .

....20

Supplementary Figure 14. Construct a model in which H_2O is located between PTA molecular layers. (a) Before optimization and (b) after optimization. (Black: carbon, yellow: oxygen, white: hydrogen)..21

Supplementary Figure 15. Effect of photocatalyst and co-catalyst dosing on photocatalytic hydrogen production. (a) Effect of different photocatalyst dosage on hydrogen evolution performance (Pt: 2 wt. %, light intensity: ~500 mW cm^{-2}). (b) Effect of different co-catalytic doses on hydrogen production performance. The mass of PTA photocatalyst is 7 mg...22

Supplementary Figure 16. The variation of PTA photocatalytic hydrogen evolution with time and its fitting parameters. (a) Using full spectrum and (b) visible light irradiation. 23

Supplementary Figure 17. Photocatalyst hydrogen production rate constant. (a) Hydrogen evolution under full spectrum (λ≥300 nm) and visible light (λ≥420 nm) conditions of PTCDA and PTA. (b) Hydrogen evolution varied with wavelength of PTA 24

Supplementary Figure 18. The stable photocatalytic HER performance of PTA under AM 1.5G, (100 mW cm^{-2}). Reaction conditions: 7.0 mg photocatalysts, 4.6 wt% Pt as co-catalysts, 100 mL, 0.2 M ascorbic acid solution and the pH is 2.45. 25

Supplementary Figure 19. Stability of PTA nanosheets was characterized by TEM. The TEM image of PTA after the cycle..26

Supplementary Figure 20. Stability of PTA nanosheets was characterized by XPS. Characterization of XPS before and after PTA nanosheets cycle. XPS analysis in (a) C 1*s*, (b) O 1*s*, and (c) Pd 4*f*. 27

Supplementary Figure 21. Optimized molecular structure. Molecular structure of (a) PTCDA and (b) PTA. 28

Supplementary Figure 22. Frontier molecular orbital. PTA (left) and PTCDA (right).....29

Supplementary Figure 23. DFT calculations of molecular frontier orbitals for PTCDA and PTA. The HOMO-LUMO gaps of PTCDA and PTA are 4.359 eV and 4.675 eV, respectively. The LUMO positions are located at -3.427 eV and -2.404 eV, respectively. 30

Supplementary Figure 24. Band structure determination. Mott-Schottky curves of (a) PTCDA and (b) PTA. Surface photovoltages of (c) PTCDA and (d) PTA......................31

Supplementary Figure 25. Energy band structure calculations. Band structure of (a) PTCDA and (b) PTA cell..33

Supplementary Figure 26. Analysis of density of states. Density of states (DOS) for (a) PTCDA and (b) PTA. (c) Contribution of O element, perylene nucleus carbon, and carbonyl carbon to conduction band minimum (CBM). Contribution: Ratio of DOS of oxygen, carbon of perylene nucleus and carbonyl carbon to total DOS integral. The range

from -5 eV to 5 eV is defined as the conduction band minimum. (d) Distribution of oxygen, perylene nuclear carbon and carbonyl carbon in PTCDA and PTA. 34

Supplementary Figure 27. Molecular dipole calculations. The dipole moment of PTCDA (left) and PTA (right) based molecular electrostatic potential. 35

Supplementary Figure 28. Schematic diagram of IEF in PTA cell. (a) Front view and (b) side view. 36

Supplementary Figure 29. Surface potential characterization. The height images (a) and the corresponding surface potential images (c) of PTCDA. The height images (b) and the corresponding surface potential images (d) of PTA. 37

Supplementary Figure 30. Zeta potential of PTCDA and PTA. Three parallel samples were tested. Each sample was tested for three times and averaged. 38

Supplementary Figure 31. The cut-off current-potential (I-V) curves of photoelectrodes under irradiation for PTA and PTCDA. Electrolyte: saturated 0.1M Na_2SO_4 aqueous solution. Under the condition of intermittent irradiation for 90 s, the applied electric field was scanned at +0.5 mV s^{-1}. 39

Supplementary Figure 32. Transient absorption spectra (TAS). (a) PTCDA and (b) PTA at different time delays after nanosecond laser excitation at 400 nm (1×10^{-5} M $_{PTA}$, aqueous solution). 40

Supplementary Figure 33. Transient absorption spectra. (a) PTA (b) PTA in $AgNO_3$ (c) PTA in ascorbic acid at different time delays after nanosecond laser excitation at 400 nm (in the form of coating film). (d) The transient absorption decay kinetics at ~650 nm of PTA and PTA in photogenerated holes and electron scavengers. 41

Supplementary Figure 34. The transient absorption decay kinetics at ~5000 nm. PTA and PTA in photogenerated holes and electron scavengers (in the form of coating film). 0.2 M ascorbic acid and $AgNO_3$ act as scavengers for holes and electrons respectively. 43

Supplementary Figure 35. Mapping of Mid-infrared TAS. Mid-infrared TAS of (a) PTCDA (b) PTA (c) PTA in $AgNO_3$ (d) PTA in ascorbic acid at different time delays after nanosecond laser excitation at 400 nm (in the form of coating film). 44

Supplementary Figure 36. The transient absorption decay kinetics at 650 nm of PTA and PTCDA (in the form of coating film). The photogenerated hole is represented at ~650 nm and the delay is slowed down for the PTA compared to the PTCDA. 45

Supplementary Figure 37. UV–vis absorption and photoluminescence spectroscopy of PTA. The excitation wavelength for photoluminescence spectroscopy is 400 nm. 46

Supplementary Figure 38. X-ray photoelectron spectroscopy (XPS) of PTA nanosheets that photo-deposited Pt and Mn_2O_3. (a) Pt 4*f* and (b) Mn 3*s* spectra acquired from PTA. 47

Supplementary Figure 39. Characterization of PTA nanosheets for photo-deposition of Pt. (a) The TEM and (b) EDS mapping. 48

Supplementary Figure 40. Characterization of PTA nanosheets for photo-deposition of Mn_2O_3. (a) TEM, (b) Mn element mapping and (c) SEM images of PTA photo-deposited Mn_2O_3. 49

Supplementary Figure 41. Structural changes in D_2O adsorption by PTCDA. ATR-FT-IR spectra of PTCDA before and after in situ adsorbing heavy water. 50

Supplementary Figure 42. Mass spectra of the reaction liquid before PTA photocatalytic hydrogen production. Reaction conditions: 7 mg photocatalyst, 0.2 M ascorbic acid (AA), 4.6 wt% cocatalyst Pt, λ > 300nm, light intensity ~530 mW cm^{-2}....................51

Supplementary Figure 43. Mass spectra of the reaction liquid. (a) After PTA photocatalytic hydrogen production. (b) A secondary mass spectrometer with m/z=191.0200. Reaction conditions: 7 mg photocatalyst, 0.2 M ascorbic acid (AA), 4.6 wt% cocatalyst Pt, λ > 300nm, light intensity ~530mW cm^{-2}....................52

Supplementary Figure 44. Synthesis method for PTA nanosheets. PTA nanosheets was prepared by facile hydrolysis-reassembly of PTCDA....................53

Supplementary Figure 45. Cross-sectional SEM image of PTA photoelectrode. PTA nanosheets were loaded onto ITO glass by drop coating to form photovoltaic electrodes. 54

2. Supplementary Notes....................55

Supplementary Note 1: Fluorescence quantum yields 55

Supplementary Note 2: The exciton binding energy of PTA 58

Supplementary Note 3: Electron mobility testing by SCLC (space charge limited current)...60

Supplementary Note 4: Estimate the yield of carrier production....................62

Supplementary Note 5: Rationalization of second-order exponential functions to fit TAS kinetic processes 65

Supplementary Note 6: Excluding the influence of morphology on the behavior of photogenerated charges....................67

Supplementary Note 7: Factors affecting the morphology of PTA nanosheets....................70

Supplementary Note 8: Explain the stability of PTA nanosheets....................72

3. Supplementary Methods....................75

Supplementary Methods 1: Photocatalytic hydrogen production unit 75

Supplementary Methods 2: Determination of internal electric fields (IEF)....................77

Supplementary Methods 3: Surface photogenerated voltage (SPV) measurement mechanism. 80

4. Supplementary Tables 81

5. Supplementary References 84

1. Supplementary Figures.

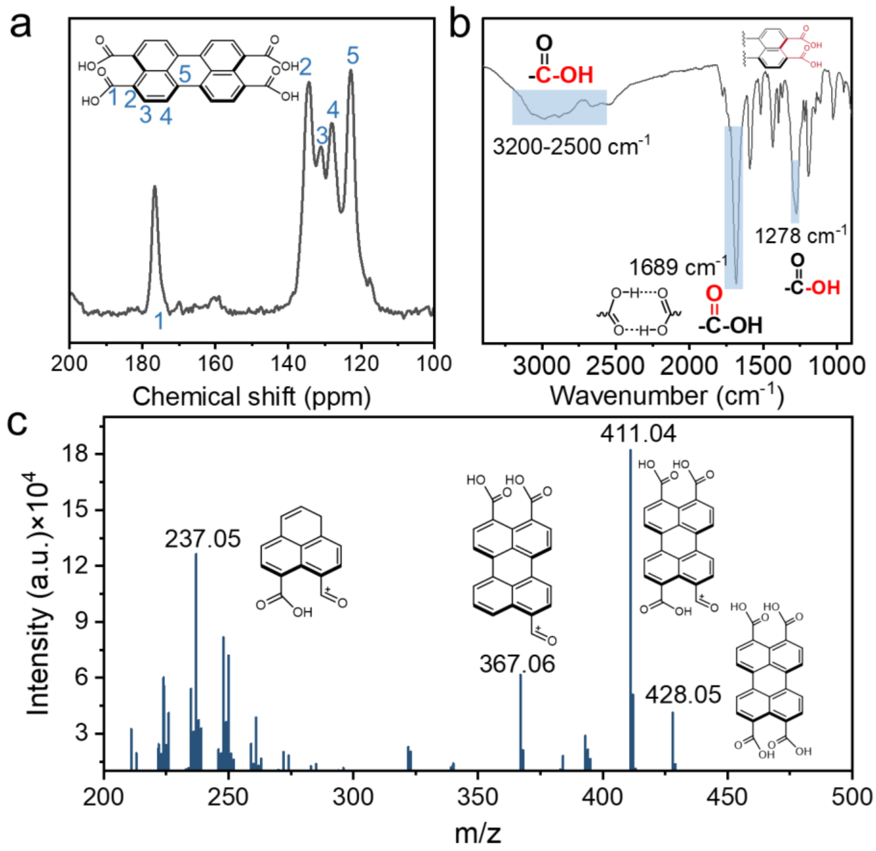

Supplementary Figure 1. Chemical structure characterization of PTA. (a) Solid 13CNMR spectrum, (b) FT-IR and (c) TOF-SIMS of PTA.

The chemical environment of carbon was characterized by solid state nuclear magnetic resonance carbon spectroscopy. The carbon on the carbonyl group (carbon 1) appears in the lowest field with a chemical shift of 176.6 ppm. Due to the conjugation effect, the chemical shift of the edge carbon (carbon 2) of perylene nucleus was 134.4 ppm. Due to the in-discriminability of the conjugations of perylene rings, the chemical shifts were 122.7-131.0 ppm (Supplementary Figure 1a). Chemical bonding was further

observed using FT-IR. Owning to the formation of hydrogen bonds between the carboxyl groups of PTA molecules, the carboxyl group peak shifted to the direction of low wave number and formed a wide and scattered peak at ~ 3200-2500 cm^{-1}. The C=O stretching vibration of free carboxylic acid is located at ~ 1760 cm^{-1}. C=O in PTA is shifted to 1689cm^{-1} influenced by hydrogen bonding, (Supplementary Figure 1b). The -OH vibration in -COOH is located at 1278 cm^{-1}. The chemical structure of perylene PTCDA with anhydride was significantly different (Supplementary Figure 3a-b). To further verify the molecular structure of PTA, Time of Fly-Secondary Ions Mass Spectroscopy (TOF-SIMS) was used to characterize the molecular weight information of PTA. An obvious PTA (m/z=428.05) was observed. Due to sputtering ionization, the PTA molecule lost -OH and produced m/z=411.04 peaks. In addition, other broken molecular fragments (m/z=367.06, 237.05) were observed. Importantly, the m/z =392.85 peak of PTCDA was hardly observed (Supplementary Figure 3c), which further explained the purity of PTA.

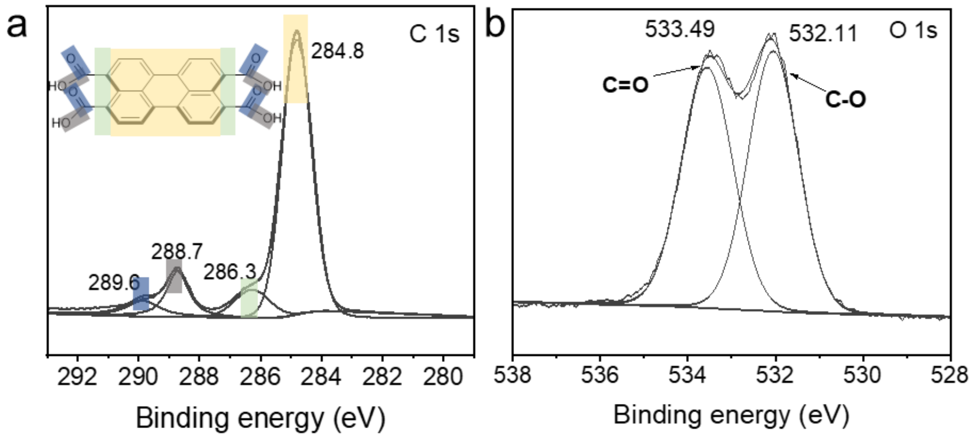

Supplementary Figure 2. X-ray photoelectron spectroscopy (XPS) analysis of PTA. (a) C 1*s* and (b) O 1*s*.

The structure of PTA was analyzed by XPS. The carboxyl group C 1s can be detected at 289.6eV. The peak of 286.3 eV represents the marginal carbon of the perylene ring, while the C 1s of perylene ring center is at 284.8eV. O1s representing carbonyl and hydroxyl groups were observed at 533.49eV and 532.11eV, respectively.

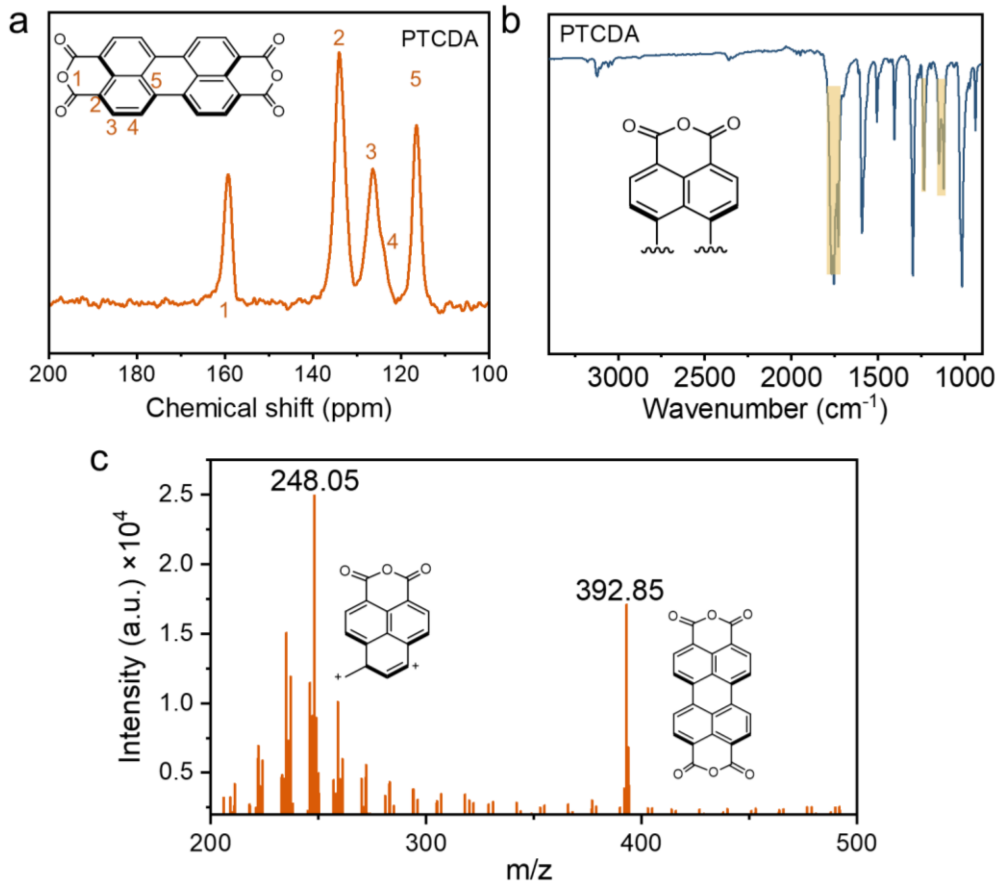

Supplementary Figure 3. Chemical structure characterization of PTA precursor PTCDA. (a) Solid ^{13}CNMR spectrum, (b) FT-IR and (c) TOF-SIMS of PTCDA.

The chemical structure of the raw material PTCDA was characterized. In the CNMR spectrum of Solid 13, 159.32 ppm was attributed to carbon (carbon 1) on the carbonyl group, and 109.0ppm^{-1}40.5ppm showed the nuclear structure of perylene. 1774cm^{-1} and 1732cm^{-1} represent the C=O peaks of anhydride.

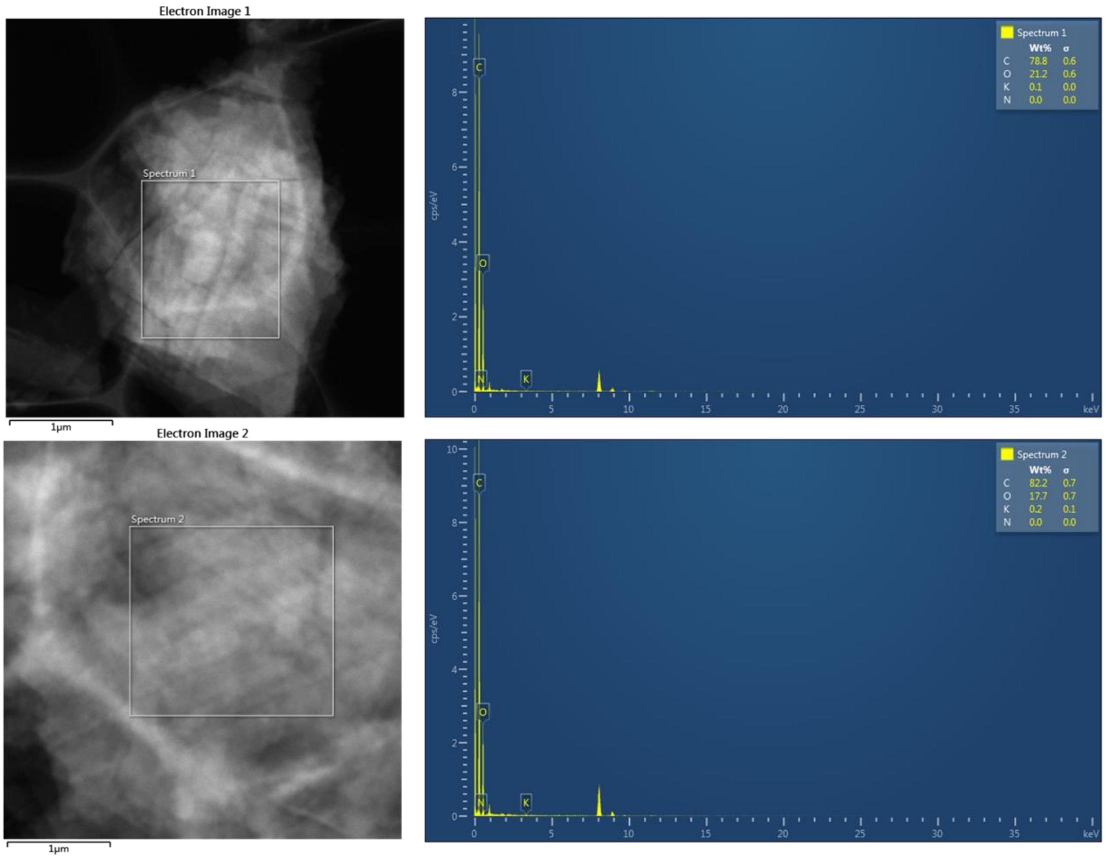

Supplementary Figure 4. Elemental analysis of two selections. TEM image with mapping data of PTA nanosheets.

Due to KOH used during the preparation of PTA, potassium may be introduced into PTA nanosheets. TEM mapping showed that there was no potassium in PTA supramolecular.

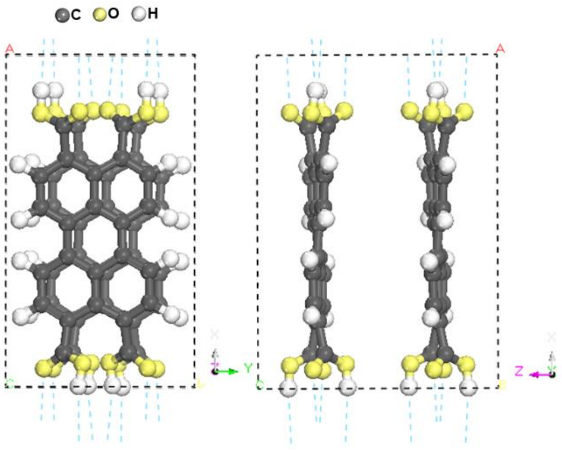

Supplementary Figure 5. Optimized PTA cell. The front view (left) and side view (right) of PTA cell.

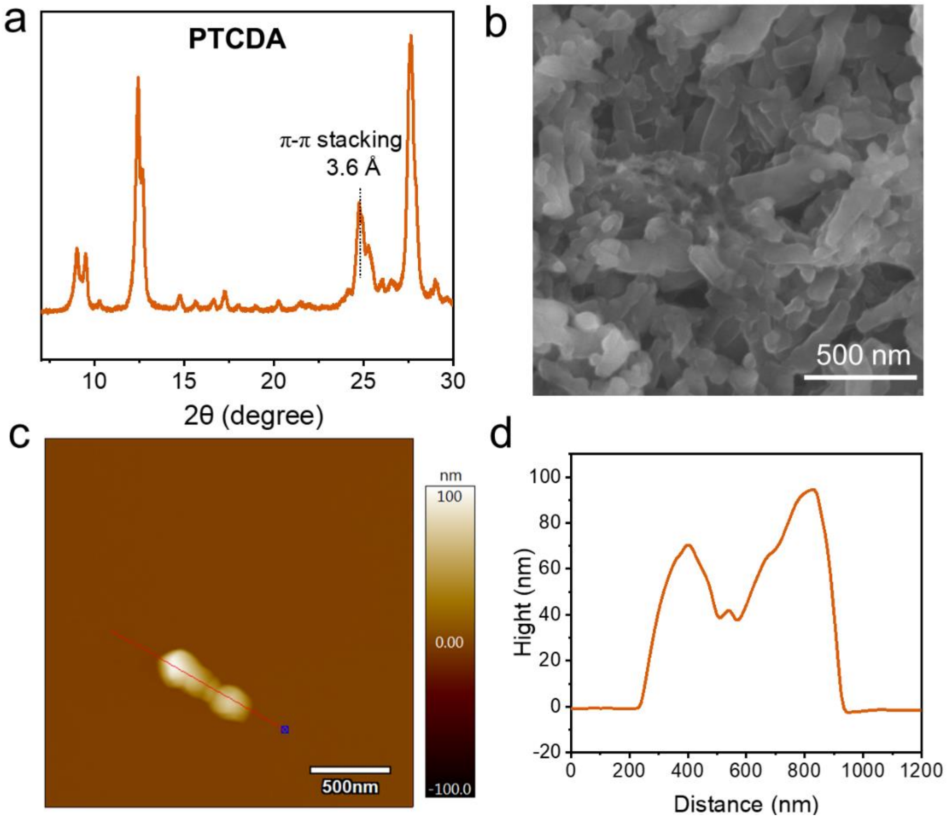

Supplementary Figure 6. The morphology and crystal structure of PTA precursor PTCDA were characterized. (a) XRD, (b) TEM, (c) AFM and (d) its height value of PTCDA.

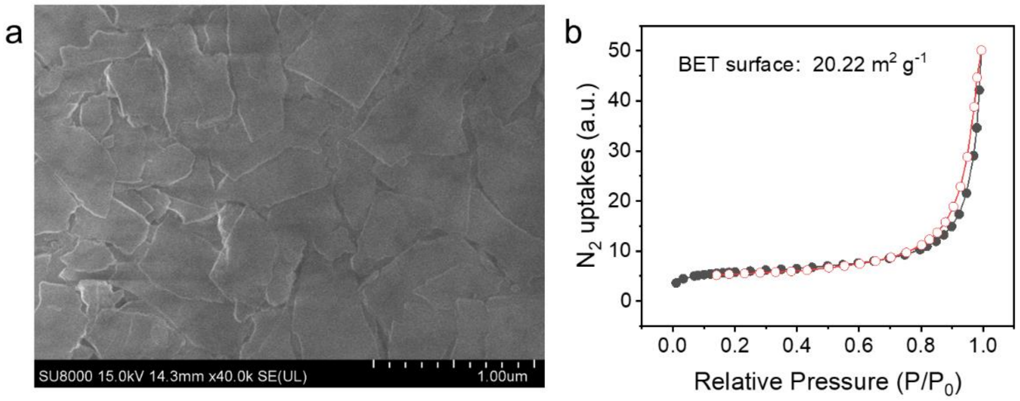

Supplementary Figure 7. Morphological characteristics of PTA nanosheets. (a) FESEM and (b) effective surface area determination (BET measurements) of PTA.

The field emission scanning electron microscopy (FESEM) image shows that the PTA has a Sheet morphology. BET indicates that the effective specific surface of these sheets area is 20.22 m^2 g^{-1}.

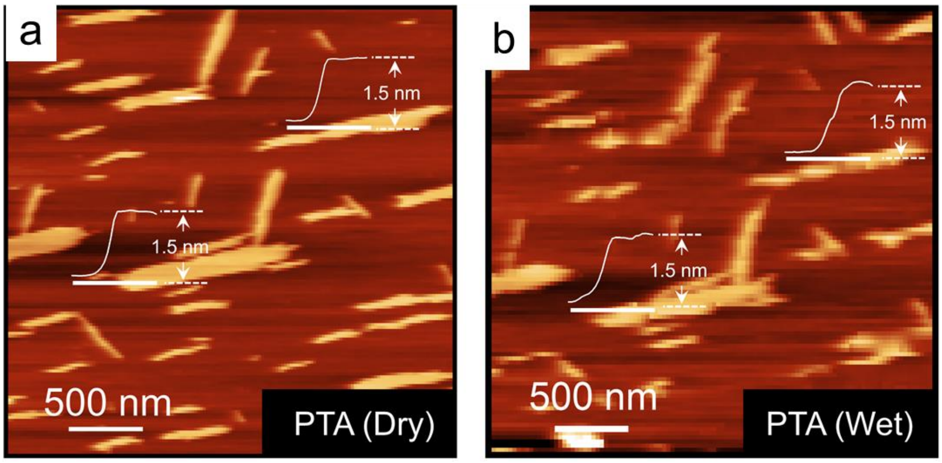

Supplementary Figure 8. Effect of water vapor on the height of PTA nanosheets. PTA nanosheets height at (a) dry-state (25% humidity) and (b) wet-state (Process in 90% humidity environment for 30min). Test conditions: Using AFM equipped with environmental control scanner, and the ambient humidity is controlled by adding water vapor in the humidity cell.

In the case of no additional water vapor is added, the humidity of the sample chamber is 25%. The AFM test in this moment is dry state. Wet-state refers to the addition of water vapor in the humidity cell. Supplementary Figure 8 (b) is the test height at 90% humidity after PTA fully absorbs water vapor for half an hour. The thickness of the PTA nanosheets in wet-state is still 1.5 nm in the humid atmosphere.

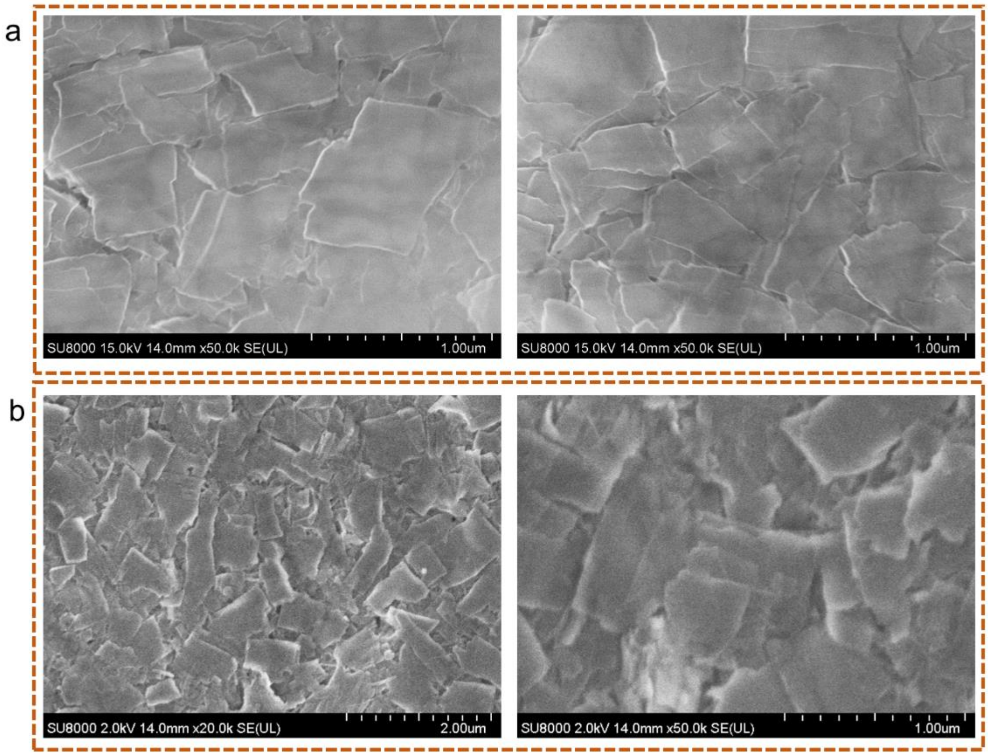

Supplementary Figure 9. Effect of water vapor on the morphology of PTA nanosheets. The SEM of (a) Dry-state and (b) wet-state of PTA (H_2O: 33000 ppm). Scan the water-absorbent sample by lowering the test vacuum (Acceleration voltage: 15kV & 2 kV).

The water content of PTA absorbed in the atomized water vapor for half an hour is about 33000 ppm. Although water causes more serious charged effect, it can still be observed that the nanosheet-like morphology of PTA has hardly changed.

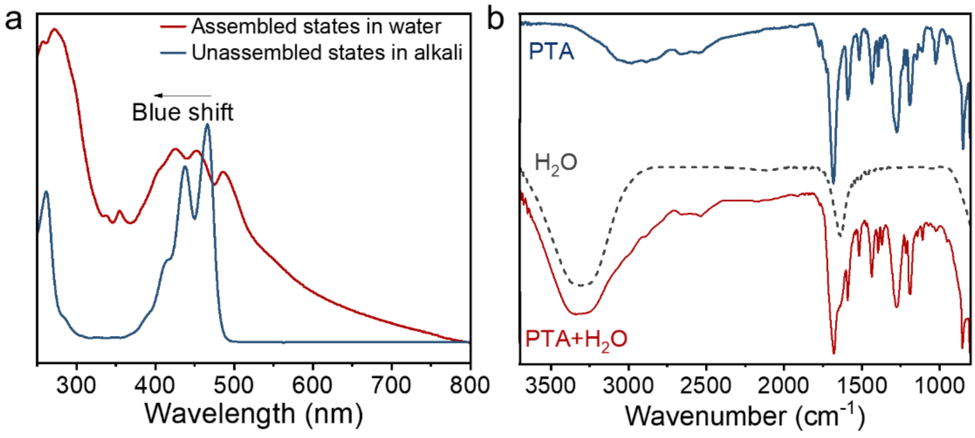

Supplementary Figure 10. Effect of water environment on the structure of PTA nanosheets. (a) UV-vis of PTA in alkali and water, respectively. (b) FT-IR of PTA adsorb water.

PTA is in a single-molecule state in alkali (Supplementary Figure 10a). In comparison, the absorption peak of PTA dispersed in water is blue-shifted. It shows that PTA is in an aggregated state in water. In-situ FT-IR data showed that the chemical structure of PTA did not change after water absorption.

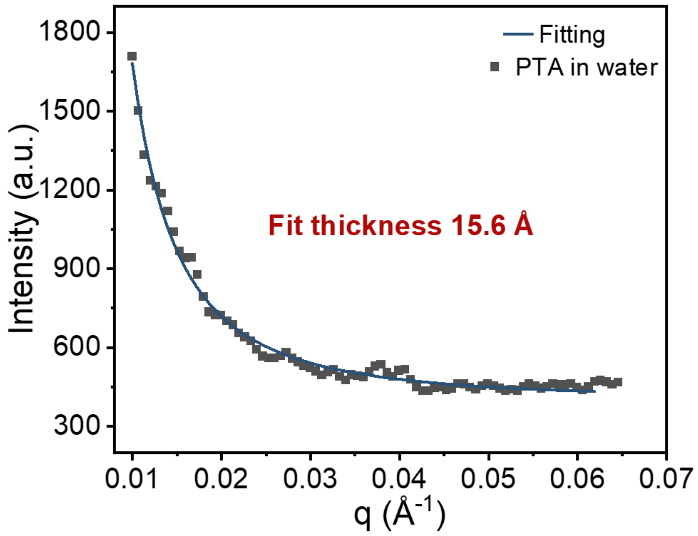

Supplementary Figure 11. Effect of water environment on the thickness of PTA nanosheets. Small Angle X-ray Scattering (SAXS) data for PTA in water (1mg mL^{-1}). (The data is fitted using lamellar model.)

The SAXS data is simulated by the lamellar model to obtain a thickness of 15.6 Å, which is highly consistent with the thickness of the dry state tested, indicating that PTA is still nanosheets in water.

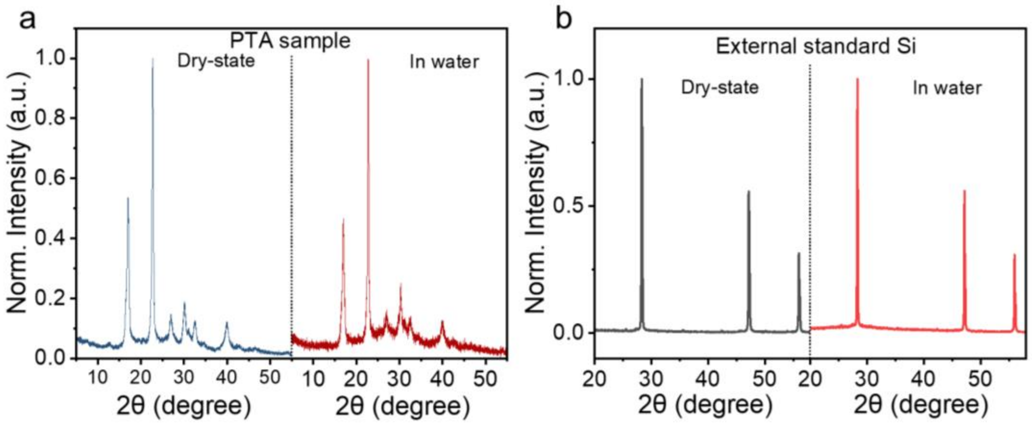

Supplementary Figure 12. Effect of water environment on the crystal structure of PTA nanosheets. X-rays diffraction (XRD) of (a) PTA sample and (b) external standard Si powder.

Compared with the XRD of dry-state, the average thickness (D) of the crystal grains perpendicular to the crystal plane calculated by the Scherer formula changes slightly when water is added (Supplementary table 1). The two main peaks change by 4% and 14%, respectively. However, Si powder as an external standard, the main peak change is greater than 11% after adding water. Therefore, it can be considered that the long-range order structure of each crystal face in water is the same as that in the dry state for PTA.

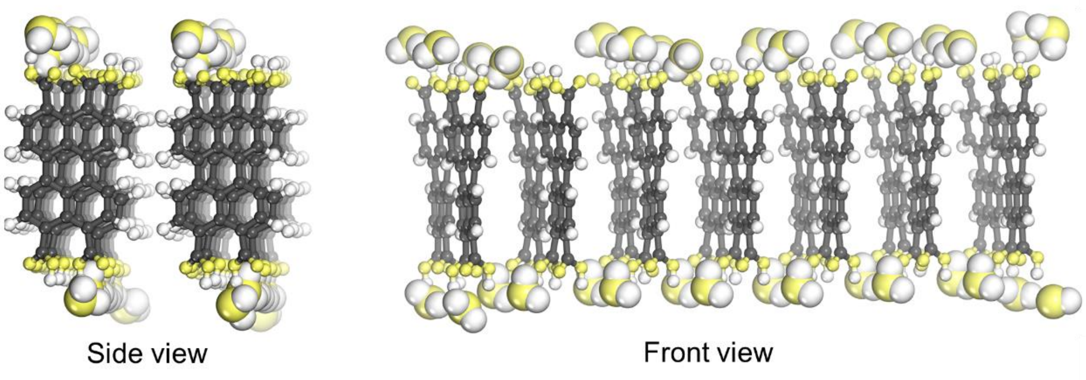

Supplementary Figure 13. Optimized PTA crystals in a water environment. Optimized model by PM6 semi-empirical method (Black: carbon, yellow: oxygen, white: hydrogen) .

Firstly, the interaction of PTA nanosheets with H_2O molecular in the water environment was explored through theoretical calculations. Under the action of the hydrogen bond between the carboxyl groups and H_2O, a hydration layer is formed around the PTA nanosheet, which effectively prevents the formation of a multilayer structure.

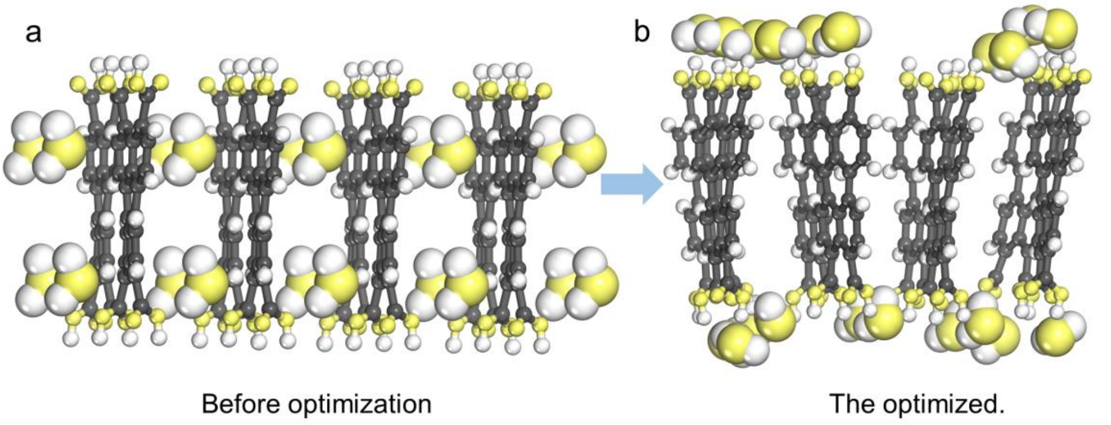

Supplementary Figure 14. Construct a model in which H_2O is located between PTA molecular layers. (a) Before optimization and (b) after optimization. (Black: carbon, yellow: oxygen, white: hydrogen)

Next, explain the reasons why the morphology of the nanosheets can be maintained in water. A model in which H_2O molecules exist between PTA molecules is constructed. After optimization, the H_2O molecules between the layers migrate to the two ends due to the hydrophilicity of carboxyl. As a result, the π-π interaction between PTA molecules is enhanced, so that the nanosheets morphology can be maintained. We further calculated that the hydrogen bonding energy between H_2O and PTA molecule is 3.78 kJ/mol. However, the π-π stacking energy between the paired PTA molecules is 46.21 kJ/mol. This proves that π-π accumulation dominates in the water environment, which is responsible for the morphology of the nanosheets.

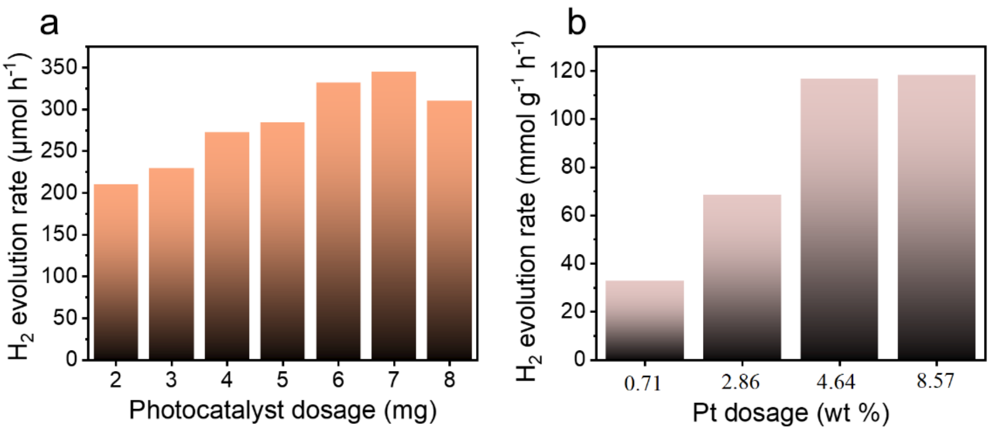

Supplementary Figure 15. Effect of photocatalyst and co-catalyst dosing on photocatalytic hydrogen production. (a) Effect of different photocatalyst dosage on hydrogen evolution performance (Pt: 2 wt. %, light intensity: ~500 mW cm^{-2}). (b) Effect of different co-catalytic doses on hydrogen production performance. The mass of PTA photocatalyst is 7 mg.

We evaluated the effect of the dosage of PTA on the photocatalytic activity and explored the amount of photocatalyst to achieve saturated light absorption[1,2]. With the increase of dosage from 6 mg to 7 mg, the actual observed HER increased from 332 μmol h^{-1} to 345 μmol h^{-1}. As the dosage increased from 7 mg to 8 mg, HER decreased from 345 μmol h^{-1} to 310 μmol h^{-1}. The reason for the decreased HER rate of large-dosage PTA photocatalyst is the light scattering and the low utilization of active sites of the photocatalyst. Therefore, the optimal dosage was 7 mg for HER[3].

Besides, it was found that the best photocatalytic performance was achieved when the mass fraction of loaded Pt was ~4.6 wt. % and the amount of catalyst was 7 mg. Too much Pt or is not conducive to the absorption of light.

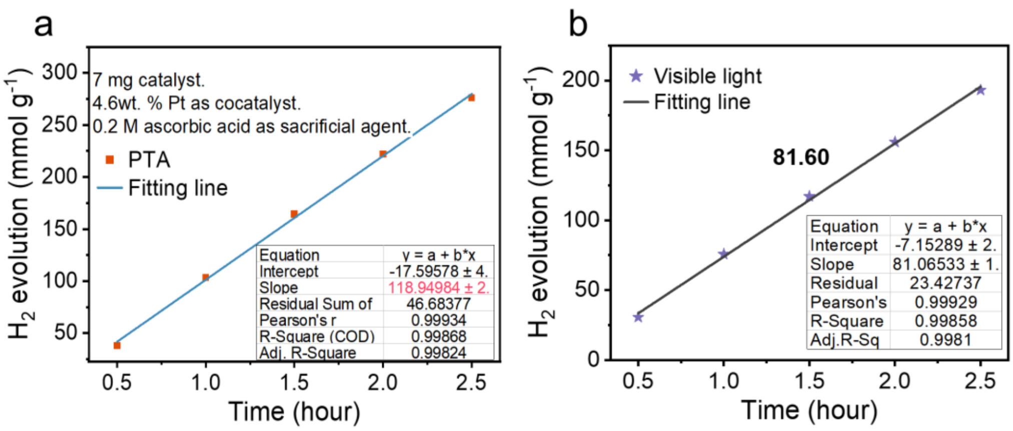

Supplementary Figure 16. The variation of PTA photocatalytic hydrogen evolution with time and its fitting parameters. (a) Using full spectrum and (b) visible light irradiation.

The amount of hydrogen in 7 mg PTA ($C_{24}H_{12}O_8$) is:

$$n(H_2)=\frac{m(\text{photocatalyst})}{M(\text{photocatalyst})}\times 6=\frac{7}{428.05}\times 6\times 1000=98.1\ \mu\text{mol} \qquad (1)$$

The amount of hydrogen produced after 2.5 h is 2080.8 umol. This value is greater than the amount hydrogen is not derived from the consumption of the photocatalyst[1]

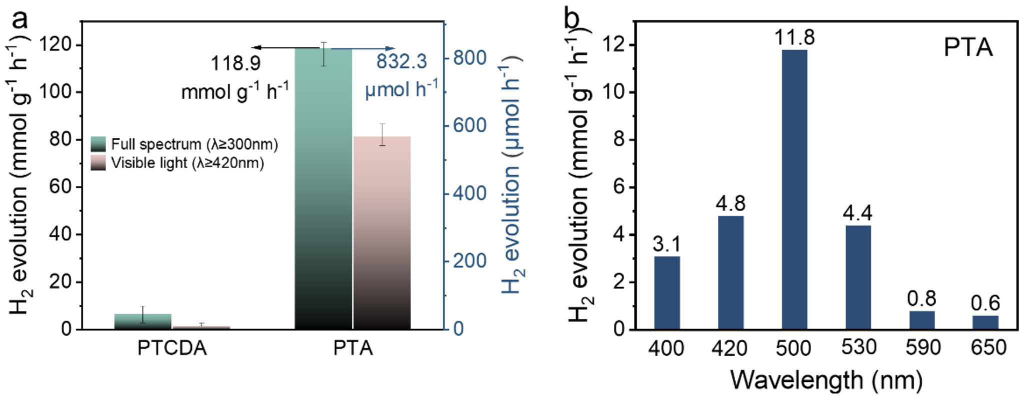

Supplementary Figure 17. Photocatalyst hydrogen production rate constant. (a) Hydrogen evolution under full spectrum (λ≥300 nm) and visible light (λ≥420 nm) conditions of PTCDA and PTA. (b) Hydrogen evolution varied with wavelength of PTA

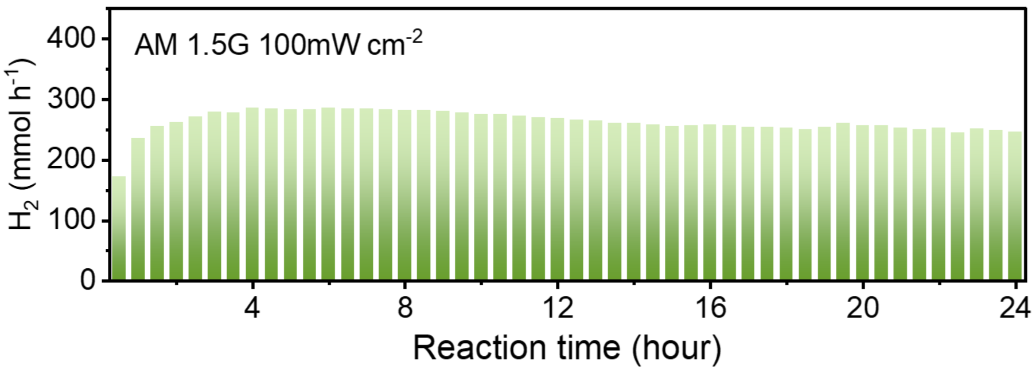

Supplementary Figure 18. The stable photocatalytic HER performance of PTA under AM 1.5G, (100 mW cm^{-2}). Reaction conditions: 7.0 mg photocatalysts, 4.6 wt% Pt as co-catalysts, 100 mL, 0.2 M ascorbic acid solution and the pH is 2.45.

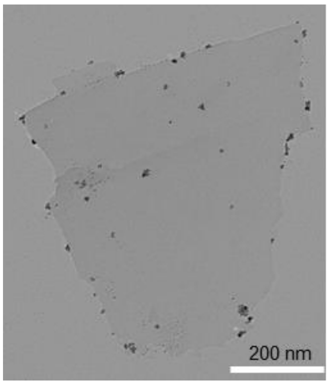

Supplementary Figure 19. Stability of PTA nanosheets was characterized by TEM. The TEM image of PTA after the cycle.

After 5 cycles, The TEM images showed that PTA still has a nanosheets structure, indicating the stability of morphology.

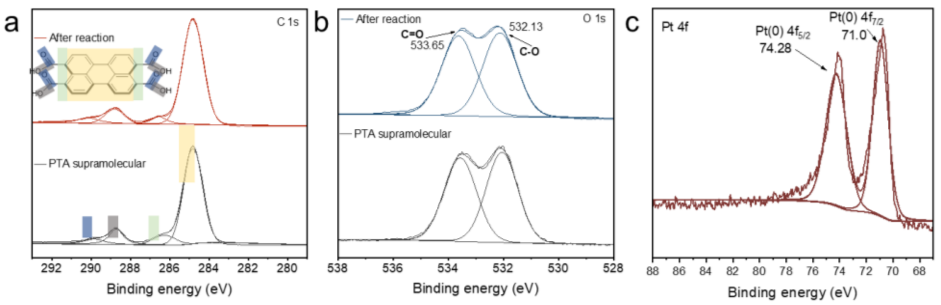

Supplementary Figure 20. Stability of PTA nanosheets was characterized by XPS. Characterization of XPS before and after PTA nanosheets cycle. XPS analysis in (a) C 1*s*, (b) O 1*s*, and (c) Pd 4*f*.

After 5 cycles, the peak positions of C1s and O1s expressed by XPS have no visible shift, indicating the stability of PTA chemical structure.

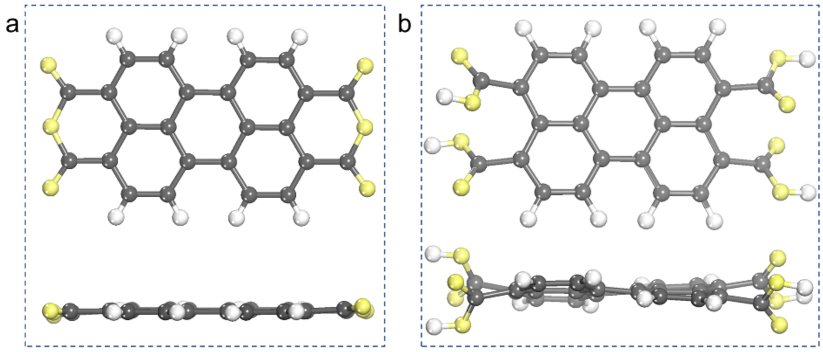

Supplementary Figure 21. Optimized molecular structure. Molecular structure of (a) PTCDA and (b) PTA.

Compared with PTCDA molecule, the presence of carboxyl group in PTA molecule distorted its plane and decrease the conjugation degree of the molecule.

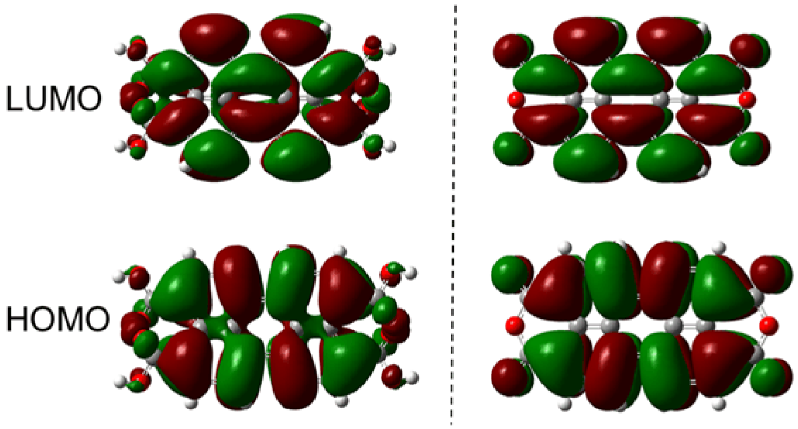

Supplementary Figure 22. Frontier molecular orbital. PTA (left) and PTCDA (right)

Compared to PTCDA, the oxygen in -OH participates in the PTA's frontier molecular orbital (HOMO and LUMO). Since the energy band of organic semiconductors is formed by the degeneracy of the energy levels of molecules, the change in energy level is consistent with the change in energy band.

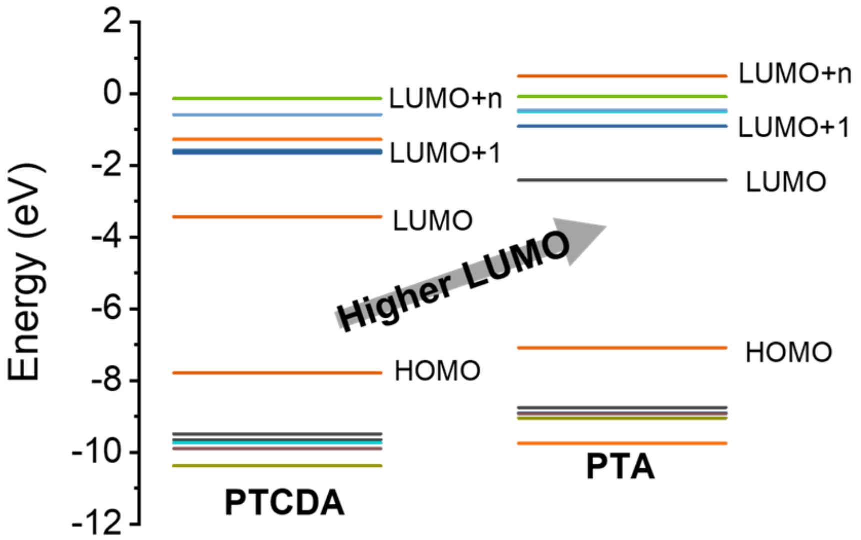

Supplementary Figure 23. DFT calculations of molecular frontier orbitals for PTCDA and PTA. The HOMO-LUMO gaps of PTCDA and PTA are 4.359 eV and 4.675 eV, respectively. The LUMO positions are located at -3.427 eV and -2.404 eV, respectively.

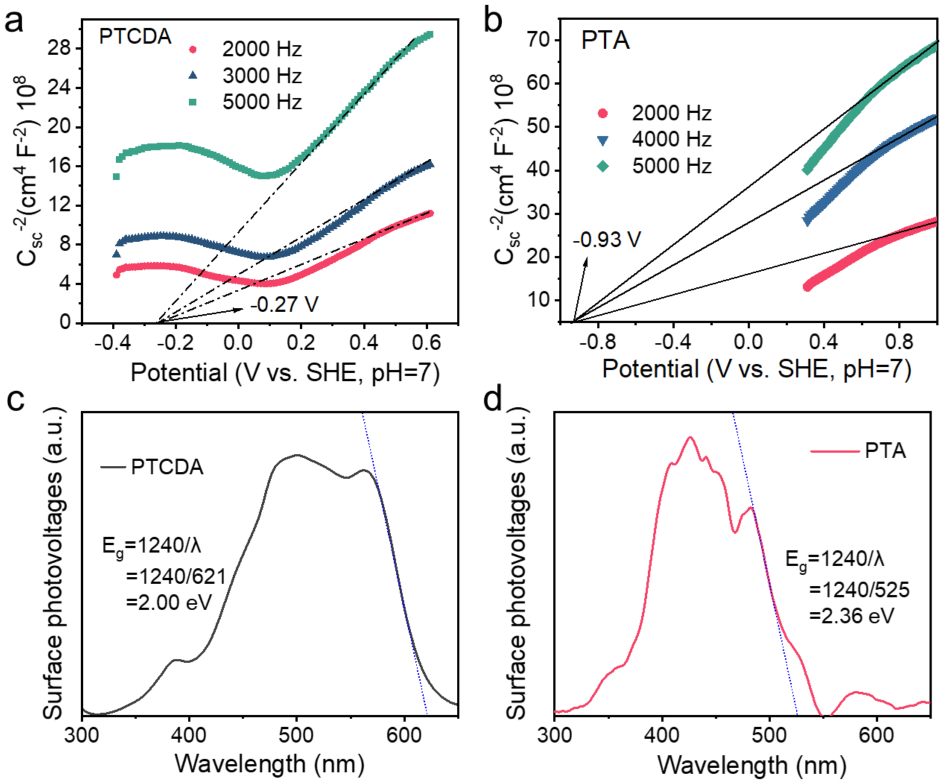

Supplementary Figure 24. Band structure determination. Mott-Schottky curves of (a) PTCDA and (b) PTA. Surface photovoltages of (c) PTCDA and (d) PTA.

Using surface photovoltage to measure optical band gap can avoid scattering effect effectively.

Band gap of PTA: E_g=2.36eV

Band gap of PTCDA: E_g=2.00 eV

The flat band potentials of PTA and PTCDA are -0.27 V and -0.93 V (vs. SHE, pH=7), respectively. Generally speaking, the position of the bottom of the semiconductor conduction band is 0.2 V negative than the flat band potential. Therefore, the conduction band positions of PTCDA and PTA are -0.47V and -1.13V (vs. SHE, pH=7),

respectively.

The position of conduction band:

PTA: E_c =-1.13 V (vs. SHE, pH=7)

PTCDA: E_c =-0.47 V (vs. SHE, pH=7)

The position of valance band (vs. SHE, pH=7):

PTA: $E_v = E_g + E_c = 2.36 + (-1.13) = 1.23$ V

PTCDA: $E_v = E_g + E_c = 2.00 + (-0.47) = 1.53$ V

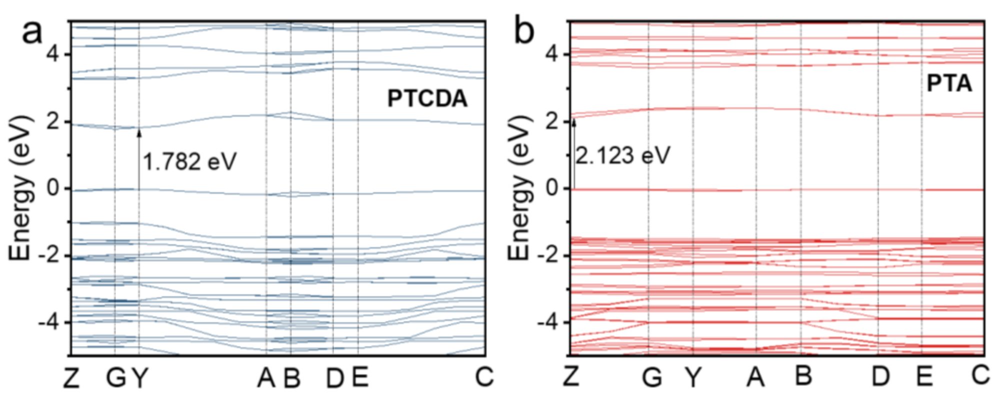

Supplementary Figure 25. Energy band structure calculations. Band structure of (a) PTCDA and (b) PTA cell.

Due to the degeneracy of molecular orbitals, continuous bands are formed with band gaps, that is, molecules self-assemble to form semiconductors. Both are direct band gap because of their valence band maximum (VBM) and conduction band minimum (CBM) are at the same K point. The band gap of PTA cell calculated theoretically is wider than that of PTCDA, which is consistent with the surface photovoltage test results.

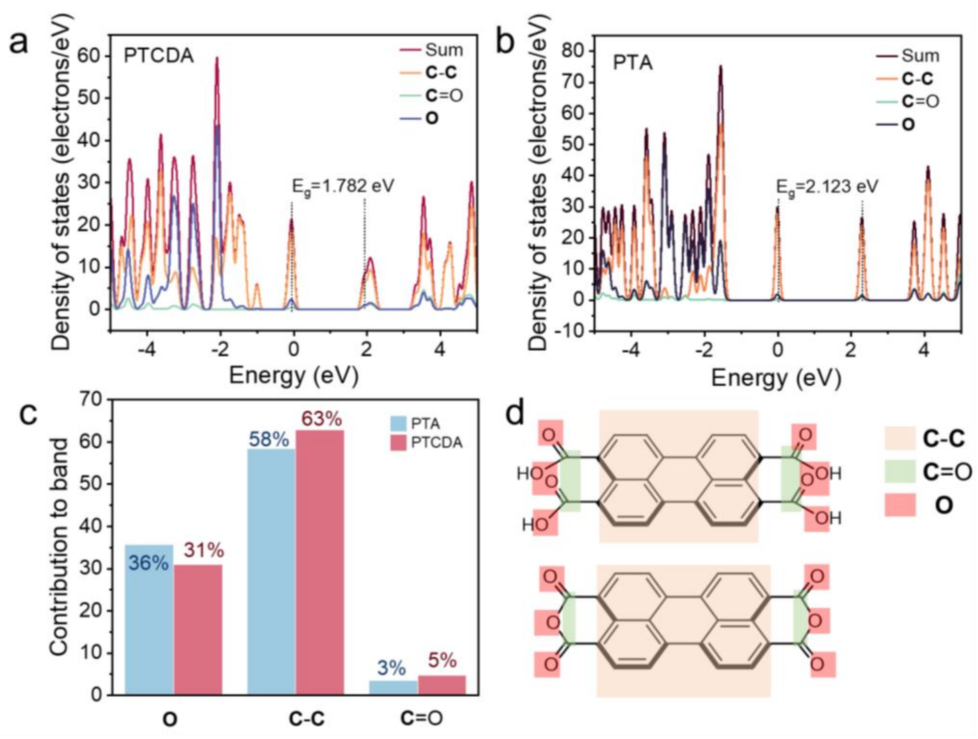

Supplementary Figure 26. Analysis of density of states. Density of states (DOS) for (a) PTCDA and (b) PTA. (c) Contribution of O element, perylene nucleus carbon, and carbonyl carbon to conduction band minimum (CBM). Contribution: Ratio of DOS of oxygen, carbon of perylene nucleus and carbonyl carbon to total DOS integral. The range from -5 eV to 5 eV is defined as the conduction band minimum. (d) Distribution of oxygen, perylene nuclear carbon and carbonyl carbon in PTCDA and PTA.

The total DOS were almost contributed by the carbon of the perylene plane, the carbon of the carbonyl group and the oxygen element. Compared with PTCDA, the contribution of C in PTA decreased, especially the C of perylene plane decreased from 63% to 58%. However, the contribution of oxygen increased from 31% to 36%. It is easy understanding since the introduction of carboxyl groups makes PTA contain more oxygen atoms.

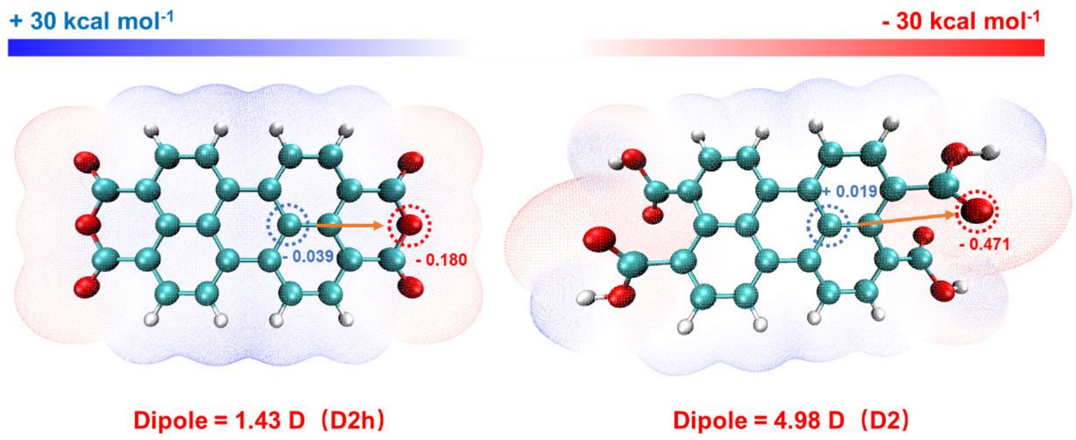

Supplementary Figure 27. Molecular dipole calculations. The dipole moment of PTCDA (left) and PTA (right) based molecular electrostatic potential.

The calculation results of the molecular electrostatic potential of PTA shows that the perylene plane has a small electrostatic potential, while the carboxyl group at the edge processes a large negative potential, thus a local dipole from the center to the edge of perylene is generated.

The dipole moment calculated by the equation of $\mu = n \times e \times d$, where μ is the net dipole moment in Debye (10^{-18} esu·cm), n is the total number of electrons, e is the charge of electron (-4.8×10^{-10} esu), and d is the difference (cm) between the “centroids” of the positive and negative charge. According to the above equation, the dipole moments of PTCDA and PTA were calculated to be 1.43 and 4.98 Debye (D), respectively. According to the symmetry of the optimized molecules (D2h for PTCDA, D2 for PTA, M062X level), we choose the charge population center reasonably.

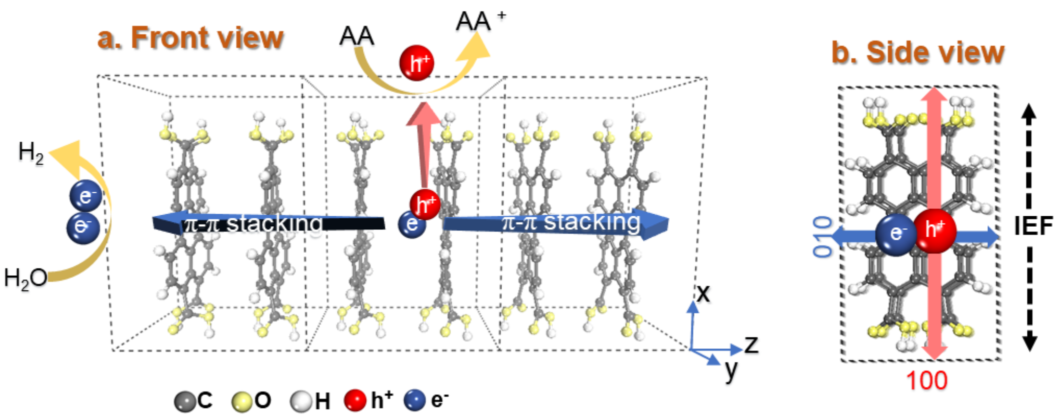

Supplementary Figure 28. Schematic diagram of IEF in PTA cell. (a) Front view and (b) side view.

Due to the ordered arrangement of PTA molecules, the local dipole accumulation of PTA molecules appears as a macroscopic IEF. The IEF facilitates the migration of photogenerated holes to the plane. And photogenerated electrons migrate to the edge via π- π stacking channels.

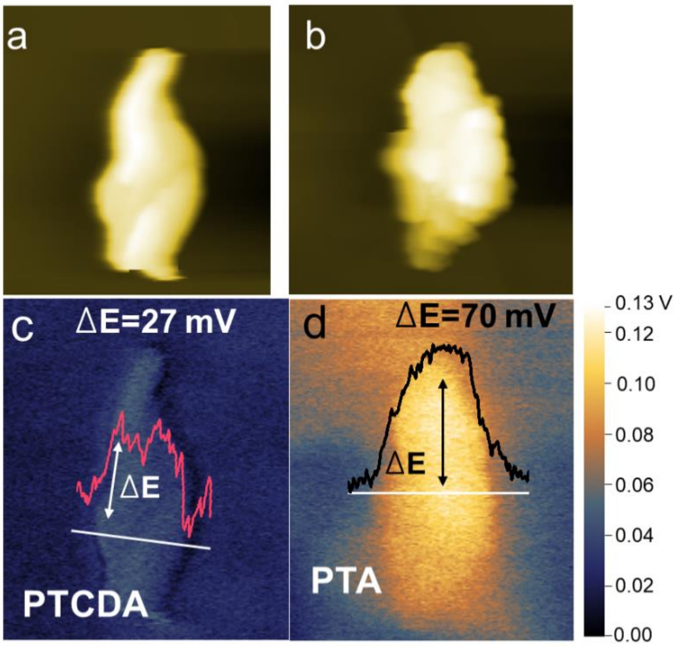

Supplementary Figure 29. Surface potential characterization. The height images (a) and the corresponding surface potential images (c) of PTCDA. The height images (b) and the corresponding surface potential images (d) of PTA.

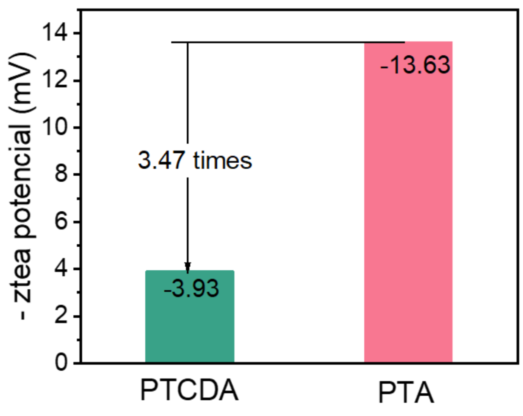

Supplementary Figure 30. Zeta potential of PTCDA and PTA. Three parallel samples were tested. Each sample was tested for three times and averaged.

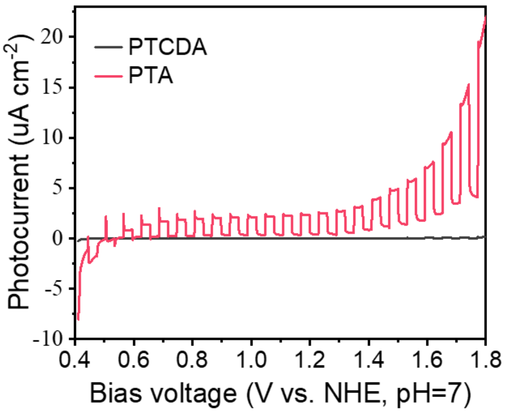

Supplementary Figure 31. The cut-off current-potential (I-V) curves of photoelectrodes under irradiation for PTA and PTCDA. Electrolyte: saturated 0.1M Na_2SO_4 aqueous solution. Under the condition of intermittent irradiation for 90 s, the applied electric field was scanned at +0.5 mV s^{-1}.

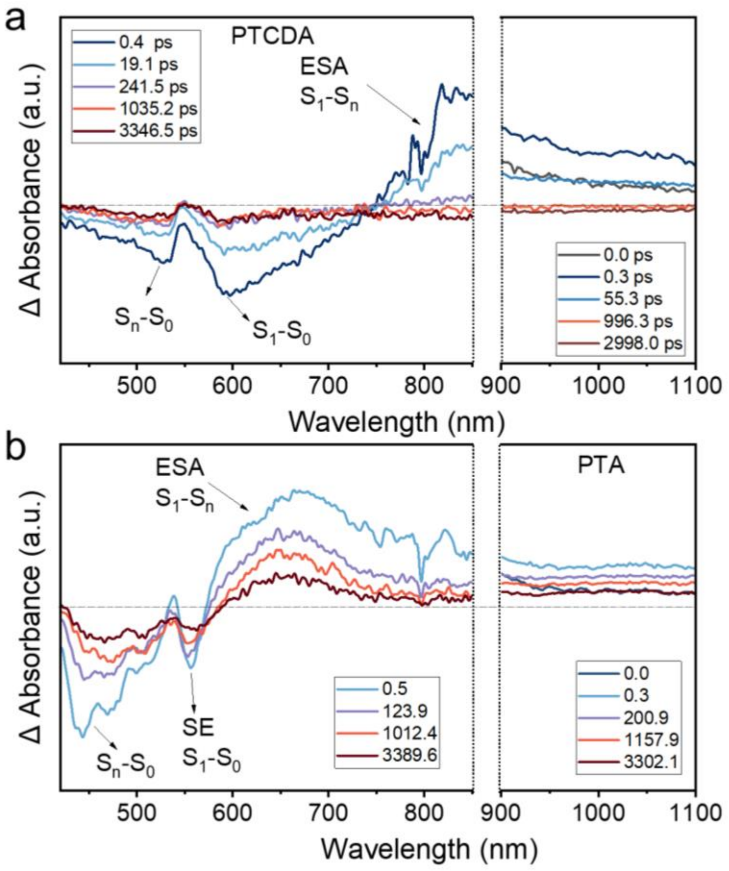

Supplementary Figure 32. Transient absorption spectra (TAS). (a) PTCDA and (b) PTA at different time delays after nanosecond laser excitation at 400 nm (1×10^{-5} M_{PTA}, aqueous solution).

The electron dynamics is detected by femtosecond-resolved transient absorption spectroscopy (TAS). The ground state bleaching (GSB) peaks at 445 nm and 477 nm correspond to steady-state absorption spectra. In stimulated emission (SE) band, a peak value of 556 nm was observed, corresponding to the steady-state fluorescence (Supplementary Figure 37). The positive absorbance represents the excited absorption of S1-Sn [4,5].For PTCDA, the excited state absorbs (ESA) has a red shift that caused by the change of band structure.

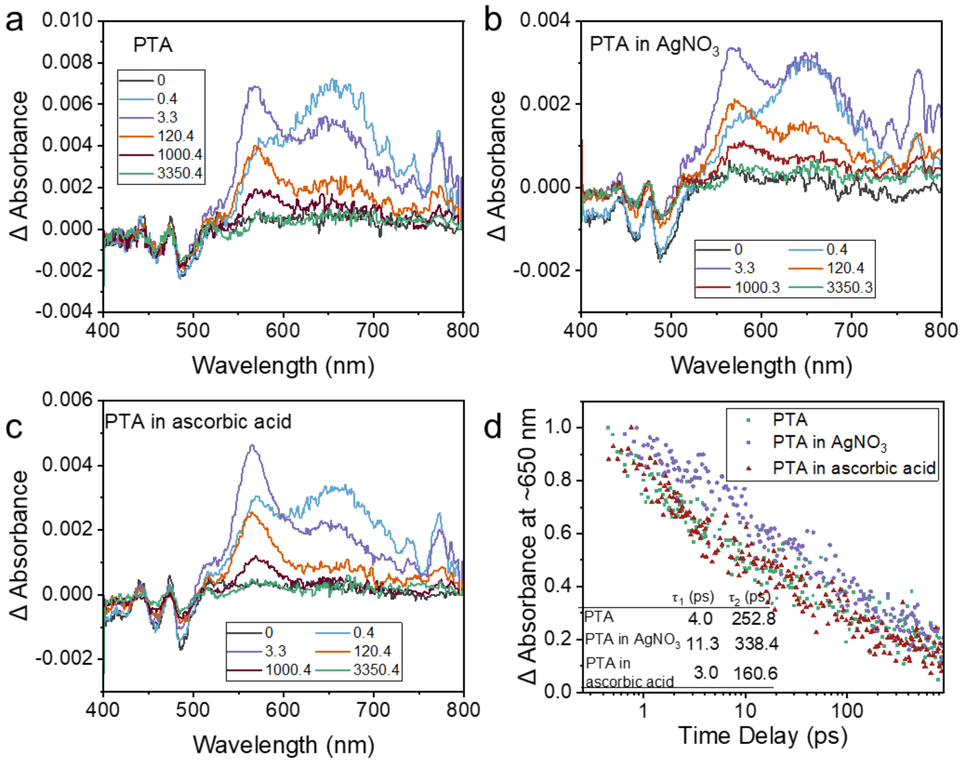

Supplementary Figure 33. Transient absorption spectra. (a) PTA (b) PTA in $AgNO_3$ (c) PTA in ascorbic acid at different time delays after nanosecond laser excitation at 400 nm (in the form of coating film). (d) The transient absorption decay kinetics at ~650 nm of PTA and PTA in photogenerated holes and electron scavengers.

First, the ESA signals representing photogenerated holes and photogenerated electrons are assigned in the TAS in the presence of hole and electron scavengers[6], respectively. The ESA signal at ~650 nm changes significantly, with a fitted decay lifetime of 252.8 ps in the absence of any scavenger (Supplementary Figure 33a), accelerating to 160.6 ps in the presence of the hole scavenger ascorbic acid (Supplementary Figure 33c), and slowing to 338.4 ps in the presence of the electron scavenger $AgNO_3$ (Supplementary Figure 33b). This validates that the ESA signal at ~650 nm can be used to analyze the

decay kinetics of photogenerated holes.

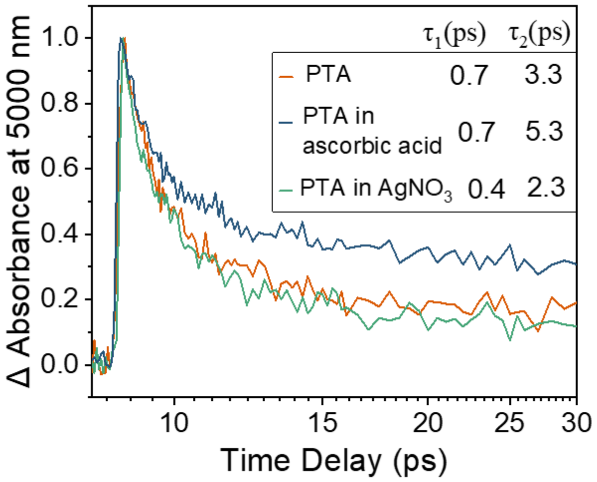

Supplementary Figure 34. The transient absorption decay kinetics at ~5000 nm. PTA and PTA in photogenerated holes and electron scavengers (in the form of coating film). 0.2 M ascorbic acid and $AgNO_3$ act as scavengers for holes and electrons respectively.

Probing the decay kinetics of photogenerated electrons in the mid-infrared region (MIR). In the absence of any scavenger, the decay time of the signal at ~5000nm was 3.3 ps, which was accelerated to 2.3 ps by the presence of $AgNO_3$ electron scavenger and further slowed to 5.3 ps by the presence of ascorbic acid hole scavenger.

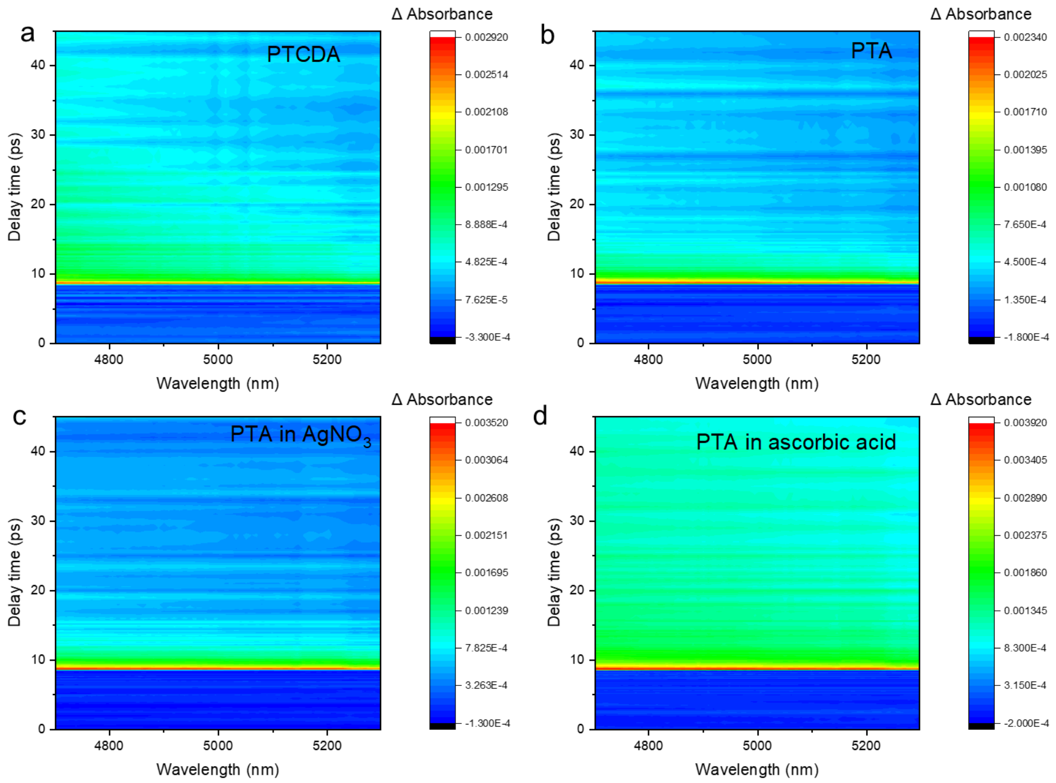

Supplementary Figure 35. Mapping of Mid-infrared TAS. Mid-infrared TAS of (a) PTCDA (b) PTA (c) PTA in $AgNO_3$ (d) PTA in ascorbic acid at different time delays after nanosecond laser excitation at 400 nm (in the form of coating film).

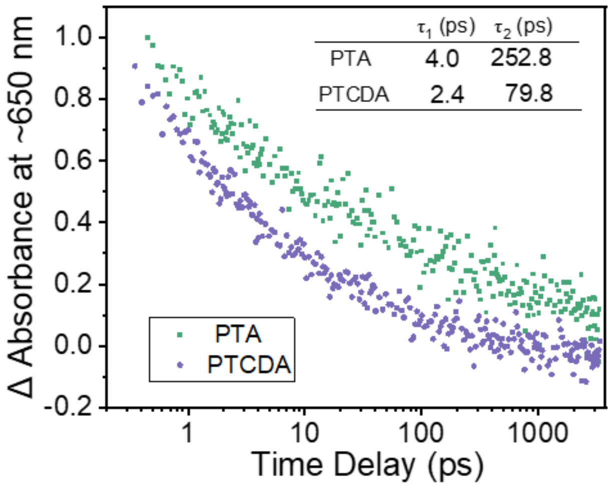

Supplementary Figure 36. The transient absorption decay kinetics at 650 nm of PTA and PTCDA (in the form of coating film). The photogenerated hole is represented at ~650 nm and the delay is slowed down for the PTA compared to the PTCDA.

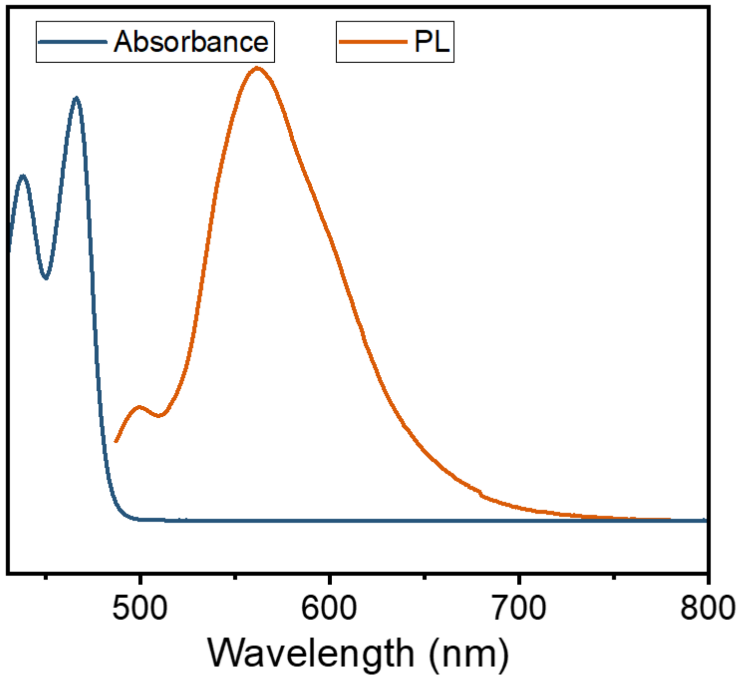

Supplementary Figure 37. UV–vis absorption and photoluminescence spectroscopy of PTA. The excitation wavelength for photoluminescence spectroscopy is 400 nm.

The steady-state absorption peaks of PTA were located at ~437nm and ~465nm. Under the excitation of λ=400nm, PTA fluoresces at ~498 nm and ~562 nm.

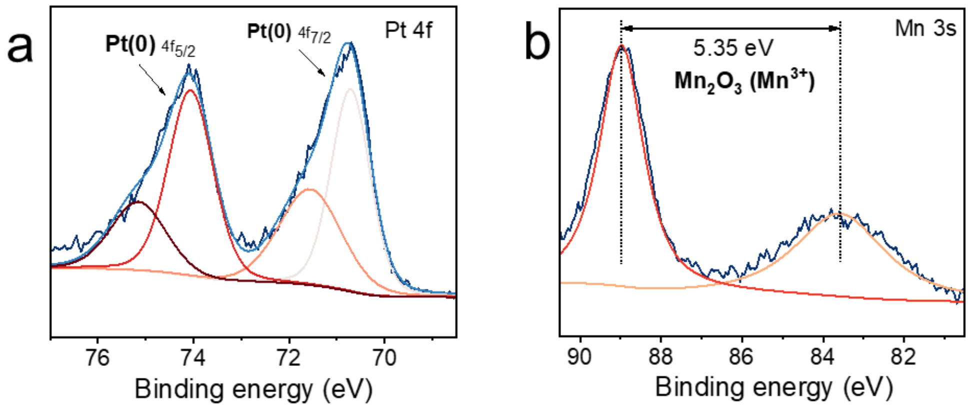

Supplementary Figure 38. X-ray photoelectron spectroscopy (XPS) of PTA nanosheets that photo-deposited Pt and Mn_2O_3. (a) Pt 4*f* and (b) Mn 3*s* spectra acquired from PTA.

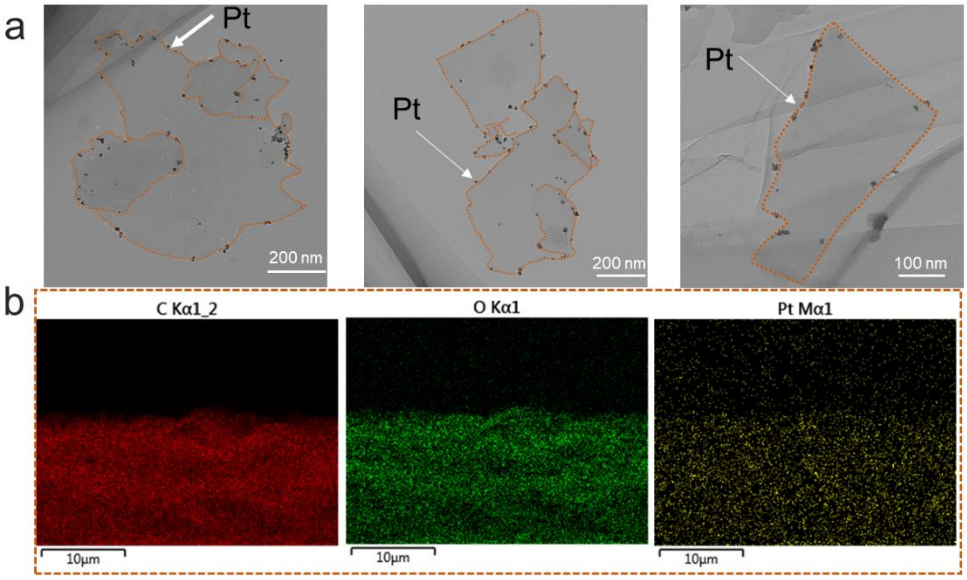

Supplementary Figure 39. Characterization of PTA nanosheets for photo-deposition of Pt. (a) The TEM and (b) EDS mapping.

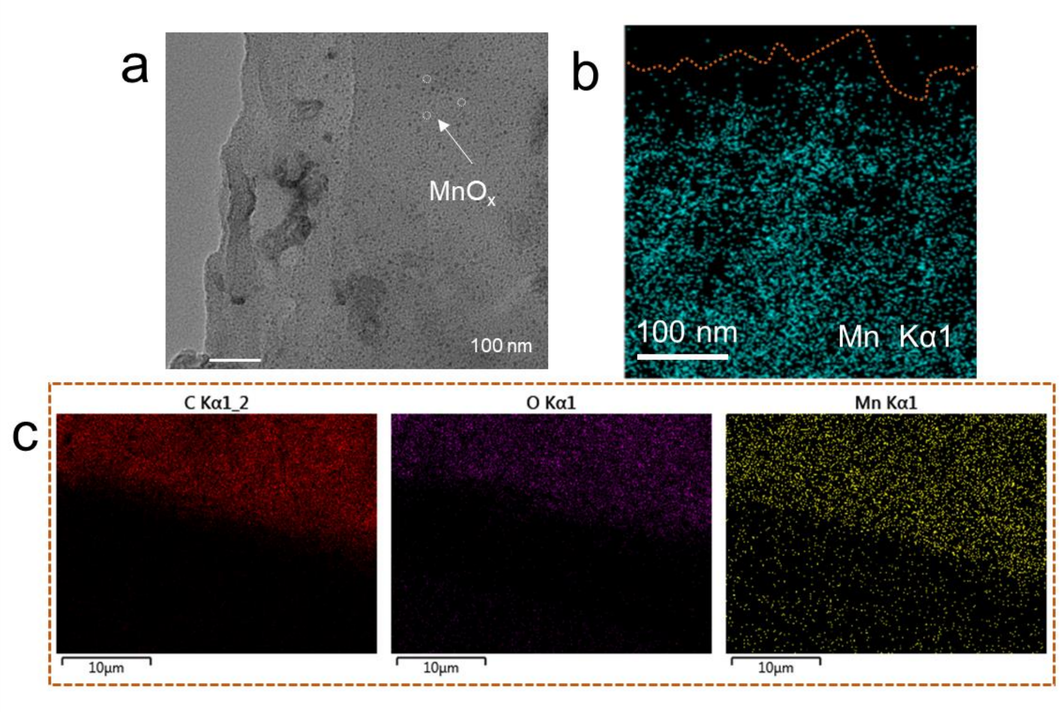

Supplementary Figure 40. Characterization of PTA nanosheets for photo-deposition of Mn_2O_3. (a) TEM, (b) Mn element mapping and (c) SEM images of PTA photo-deposited Mn_2O_3.

TEM and SEM images showed that Mn_2O_3 was deposited uniformly on the plane of PTA (that is (100) facets).

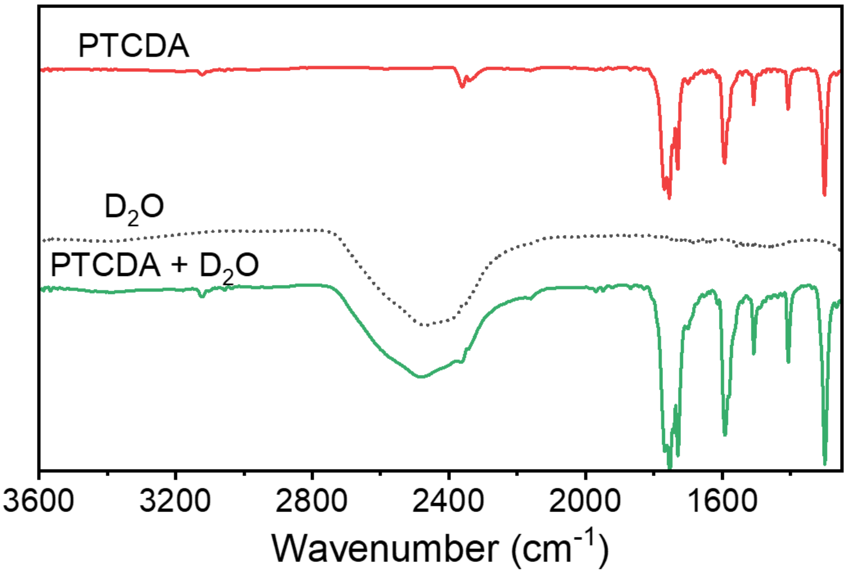

Supplementary Figure 41. Structural changes in D_2O adsorption by PTCDA. ATR-FT-IR spectra of PTCDA before and after in situ adsorbing heavy water.

There was no significant change in FT-IR of PTCDA after D_2O adsorption, indicating that the hydrophilic sites of PTCDA may be few.

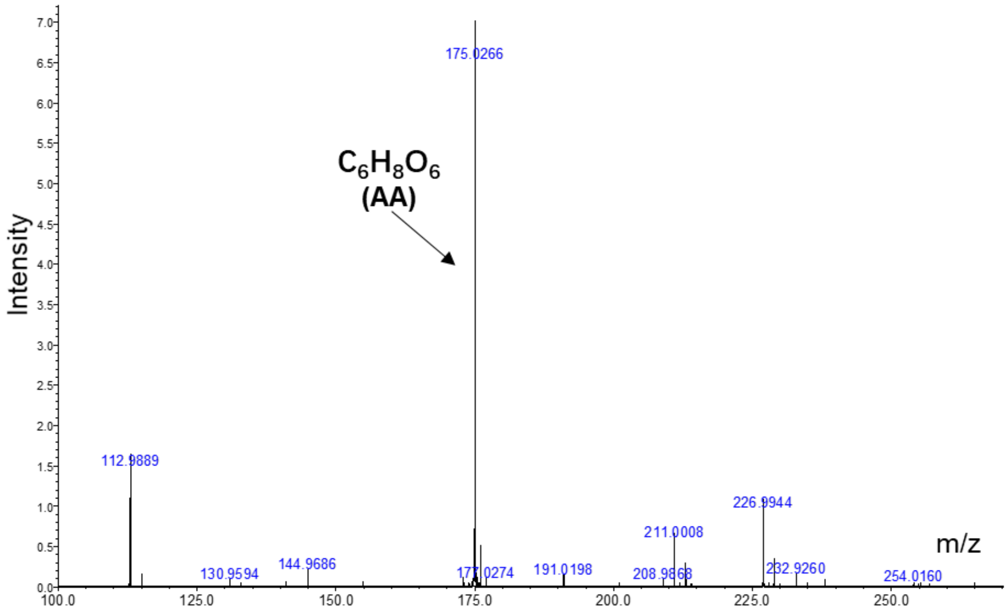

Supplementary Figure 42. Mass spectra of the reaction liquid before PTA photocatalytic hydrogen production. Reaction conditions: 7 mg photocatalyst, 0.2 M ascorbic acid (AA), 4.6 wt% cocatalyst Pt, λ > 300nm, light intensity ~530 mW cm^{-2}.

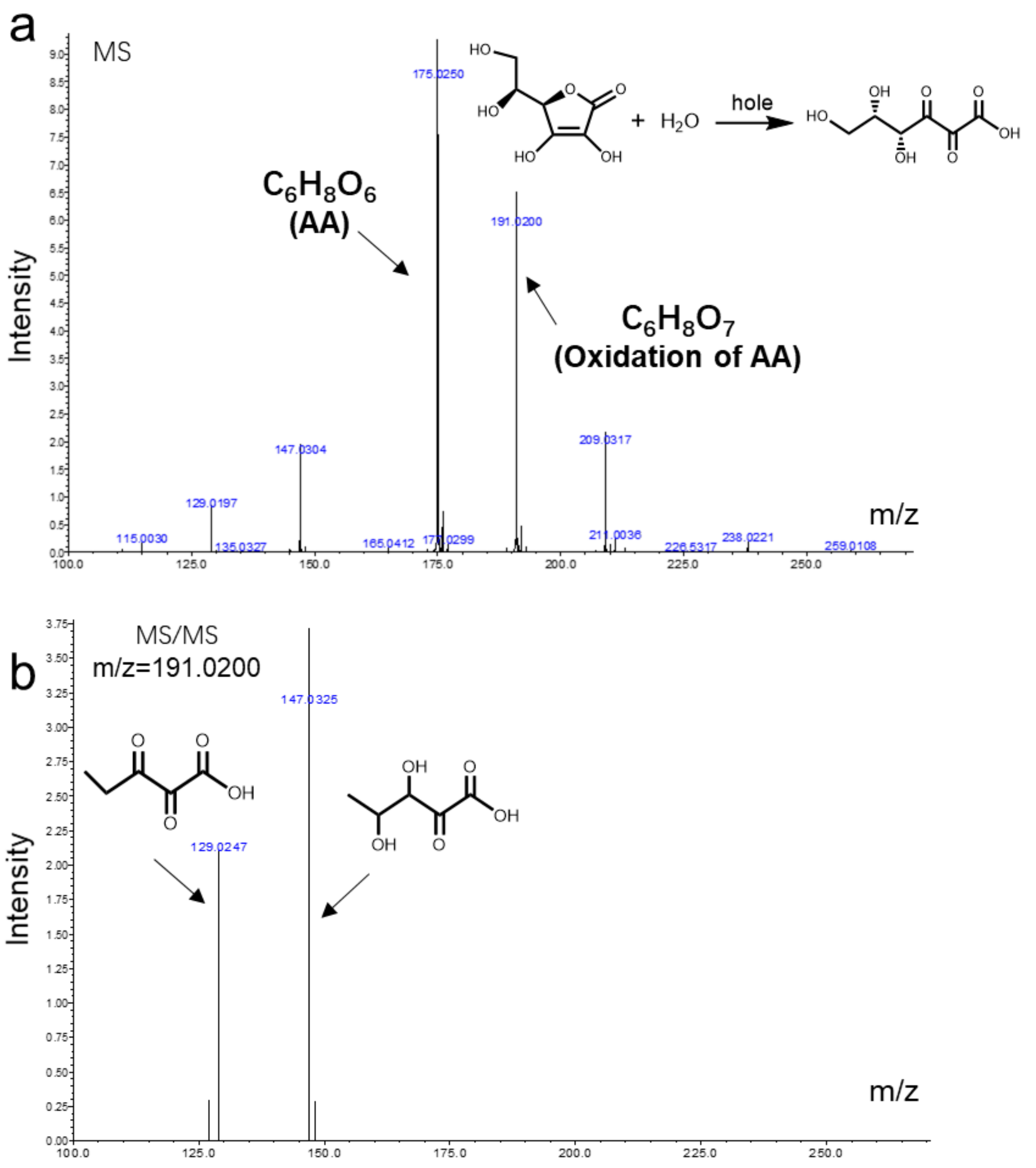

Supplementary Figure 43. Mass spectra of the reaction liquid. (a) After PTA photocatalytic hydrogen production. (b) A secondary mass spectrometer with m/z=191.0200. Reaction conditions: 7 mg photocatalyst, 0.2 M ascorbic acid (AA), 4.6 wt% cocatalyst Pt, λ > 300nm, light intensity ~530mW cm^{-2}.

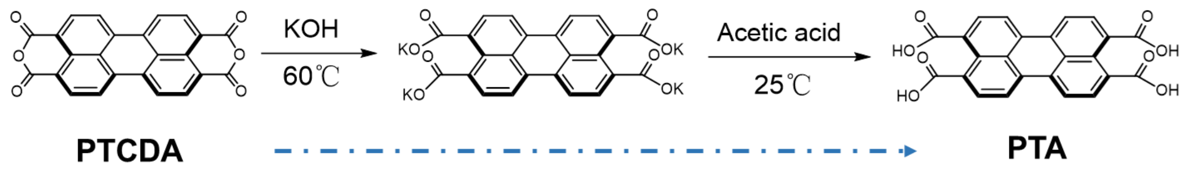

Supplementary Figure 44. Synthesis method for PTA nanosheets. PTA nanosheets was prepared by facile hydrolysis-reassembly of PTCDA.

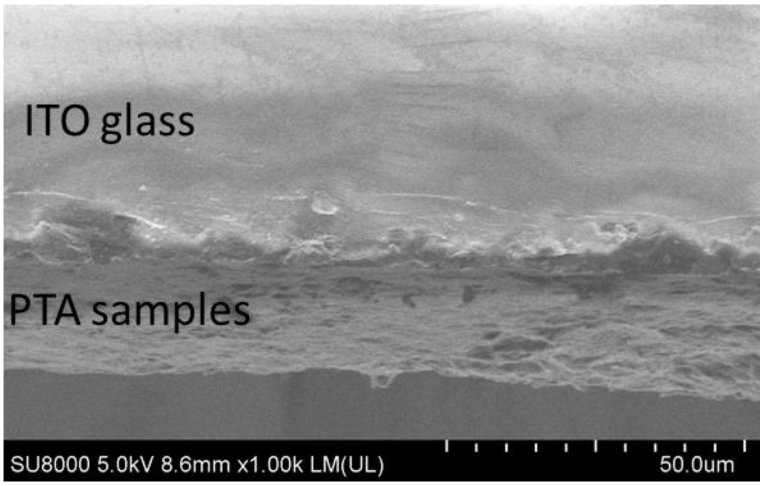

Supplementary Figure 45. Cross-sectional SEM image of PTA photoelectrode. PTA nanosheets were loaded onto ITO glass by drop coating to form photovoltaic electrodes.

2. Supplementary Notes

Supplementary Note 1: Fluorescence quantum yields

Relative quantum yield calculations were performed using a modified procedure by Williams et al[7]. The quantum yield of a sample relative to a reference compound can be calculated using the following equation:

$$Q_S = Q_R\left(\frac{A_R}{A_S}\right)\left(\frac{E_S}{E_R}\right)\left(\frac{I_R}{I_S}\right)\left(\frac{n_S^2}{n_R^2}\right) \quad (2)$$

Q_S - Quantum yield of the sample

Q_R - The known quantum yield of the reference

A_R - Absorption of the reference at a single excitation wavelength

A_S - Absorption of the sample at a single excitation wavelength

E_R - The combined emission of the reference in its emission range

E_S - The combined emission of the sample over its emission range

I_R - The intensity of the excitation wavelength of the reference

I_S - The intensity of the excitation wavelength of the sample

n_R - Refractive index of the solvent of the reference

n_S - Refractive index of the solvent of the sample

If the excitation wavelengths of the sample and the reference are the same, the equation simplifies to:

$$Q_S = Q_R\left(\frac{A_R}{A_S}\right)\left(\frac{E_S}{E_R}\right)\left(\frac{n_S^2}{n_R^2}\right) \quad (3)$$

This calculation can be done using a single concentration, or a range of concentrations.

We use the slope of the integrated emission intensity versus absorbance curve at the excitation wavelength, avoiding errors in individual measurements.

$$Q_S = Q_R\left(\frac{Slope_S}{Slope_R}\right)\left(\frac{n_S^2}{n_R^2}\right) \quad (4)$$

We used the reported QY = 0.94 for rhodamine 6G in ethanol as a control compound. Typically, fluorescence and absorbance measurements were made by injecting 3.5 mL of the solution into a 10 mm quartz cuvette. Absorbance at 488 nm was tested. The emission spectra were collected by excitation at 488 nm and the fluorescence peaks in the 500-700 nm range were integrated.

To minimize the effect of self-absorption effects, a lower concentration of the substance to be measured was used. An ethanolic solution of 0.02-0.1 mmol/L of rhodamine 6G (n = 1.361) was used as a reference. The 6.7-33.4 umol/L of PTA water (n = 1.333) dispersion was used as the sample to be tested. The quantum yields for PTA in water were 4.9%.

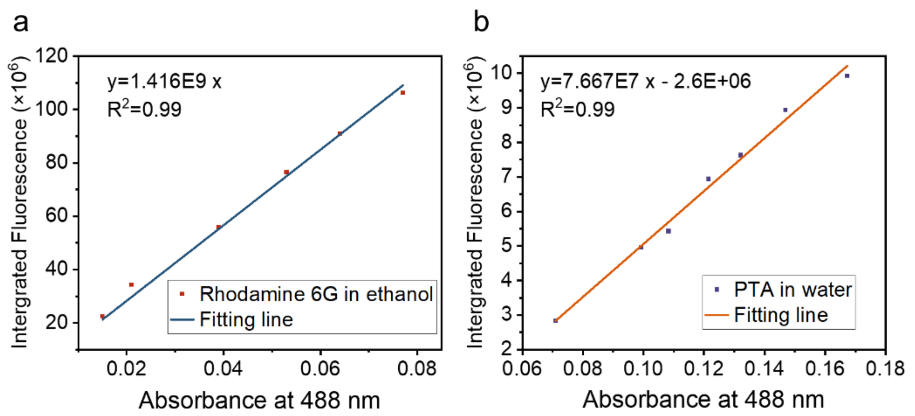

Supplementary Figure 46: Fluorescence integrated area versus absorbance curves. (a) reference rhodamine 6G in ethanol and (b) PTA in water. The solutions were excited at 488 nm and the spectra were collected between 500-700 nm. The estimated slopes used for quantum yield calculations are shown on each graph. The quantum yields for PTA in water were 4.9%.

Supplementary Note 2: The exciton binding energy of PTA

The Coulomb attraction energy of electrons and holes is used to evaluate the exciton binding energy of PTA through the Coulomb equation as follows [8,9]:

$$E_c = \iint \frac{\rho^{hole}(r_1)\rho^{ele}(r_2)}{|r_1 - r_2|} dr_1 dr_2 \quad (5)$$

Where ρ represents the densities of the natural transition orbitals (NTOs) of the hole ρ^{hole} and the electron ρ^{ele}, respectively. The NTOs are derived by the Cubgen tool included in the Gaussian 09 suite.

Based on the time-dependent density functional theory (TD-DFT), the main transitions of the first five singlet excited states were determined by the vibrator strength. Exciton binding energy analysis was performed on PTA with different *π-π* stacking layers (Supplementary Figure 47). As expected, with the increase in the number of layers, the charge transfer excitation process is subsequently enhanced and the exciton binding energy decreases from 4.41 eV to 0.72 eV. At lower exciton binding energies, excitons dissociate more readily into photogenerated electrons and photogenerated holes.

Further, when the number of layers is 6, the electrons of the PTA stacker are mainly enriched on the perylene, while the holes are enriched on the carboxyl group of the intermediate PTA molecule (Supplementary Figure 48). This further validates the IEF-induced carrier behavior in the PTA molecule, i.e., holes migrate to the carboxyl group driven by the IEF, while electrons migrate to the edges of the nanosheets through channels formed by the *π-π* stacking.

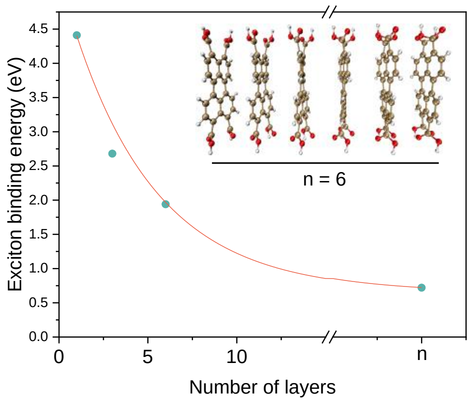

Supplementary Figure 47. Exciton binding energy of PTA in different layer stacking. Trend of exciton binding energy from monolayer to infinite layer calculated by time-dependent density functional theory (TD-DFT).

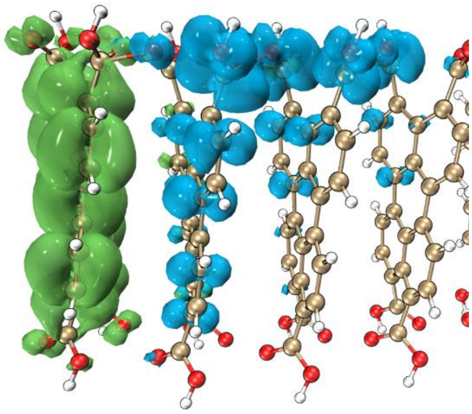

Supplementary Figure 48. Excited state electron-hole distribution of PTA stack (layer number 6). Green equivalence plane represents electron distribution; blue equivalence plane represents hole distribution.

Supplementary Note 3: Electron mobility testing by SCLC (space charge limited current)

To evaluate the charge mobility, the electron mobility of PTCDA and PTA was measured by SCLC method [10]. Its J-V curve is shown in Supplementary Figure 51. The mobility values were calculated by the Mott–Gurney law for the sandwich-type device:

$$\mu=\frac{8JL^3}{9\varepsilon_0\varepsilon_r V^2} \quad (6)$$

Where J, ε_r, ε_0 and L represent the current density, relative permittivity, the vacuum permittivity and the distance between the two electrodes, respectively. It is assumed that ε_r=3, and ε_0 is the same for all crystals and a value of 8.85×10^{-14} has been used from previous reports [11]. The electron mobility is calculated to be 1.1 and 3.2 $cm^2\ V^{-1}\ s^{-1}$ for PTCDA and PTA, respectively. For PTA nanosheets, the enhanced internal electric fields and the long-range ordered structure contribute to their electron mobility.

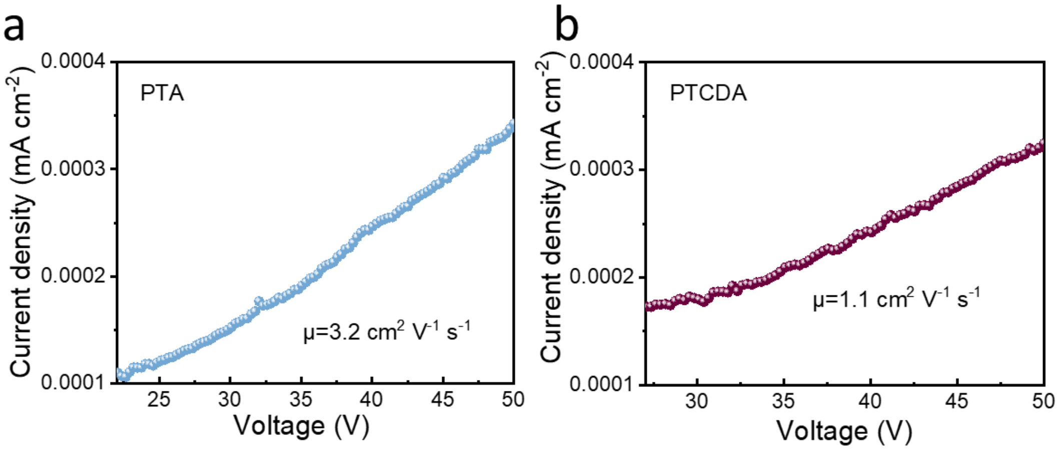

Supplementary Figure 49. Electron mobility testing by space-charge limited current (SCLC). The plot of the current density (J) versus applied voltage (V) measured in a ~200 μm and ~1.5 cm^2 area thick (a) PTA and (b) PTCDA samples at

room temperature in air.

Supplementary Note 4: Estimate the yield of carrier production

The observed rate constant in PTA nanosheets is: $1/\tau = k_1 + k_2$

Where τ is the photogenerated electron lifetime from TAS data, 134.2 ps.

k_1 is the rate of monomer decay, $k_1=1/\tau_1$, τ_1 is the stimulated emission (SE) delay from TAS data, 1421.9 ps. k_2 is the charge transfer rate constant, it is further calculated that $k_2=0.006748\ ps^{-1}$.

And then computing the efficiency carrier production (Q):

$$Q=\frac{k_2}{k_1+k_2}\times 100\%=90.6\% \quad (7)$$

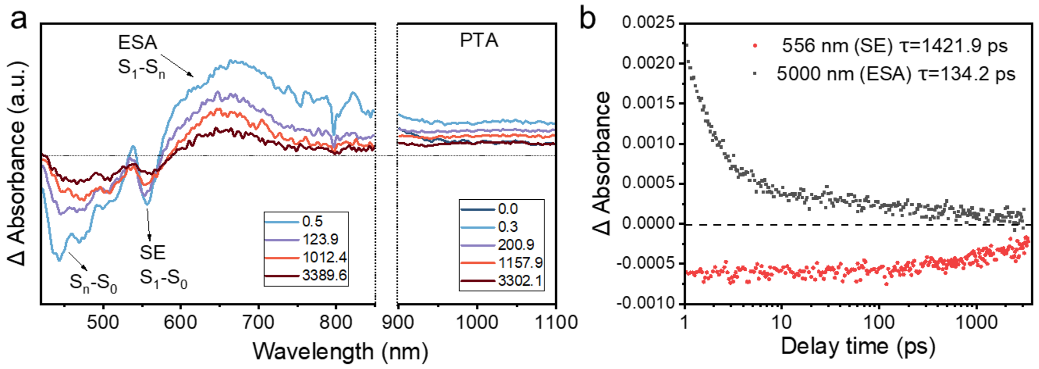

Supplementary Figure 50. Estimate the yield of carrier production. (a) TAS of PTA at different time delays after nanosecond laser excitation at 400 nm (1×10^{-5} M, aqueous solution). (b) transient absorption decay kinetics at 5000 nm and 556 nm of PTA.

As shown in Figure 2c in manuscript, the apparent quantum efficiency of hydrogen production of the PTA nanosheets at 400 nm excitation was 4.8%, which is lower than the estimated carriers generation rate (90.6%). The significant difference between the

apparent quantum efficiency of hydrogen production and the carrier hydrogen production rate can be explained as follows:

On the one hand, the carriers produced were not fully used for hydrogen production, which may be attributed to processes such as carriers are used to generate reactive oxygen species. As shown in the electron paramagnetic resonance (EPR) measurement (Supplementary Fig.51), where the photogenerated electrons of PTA are trapped by dissolved oxygen to form $\cdot O_2^-$ in an air atmosphere. It further reacts with H^+ to form $\cdot OH$.

On the other hand, in general, after photoexcitation of a semiconductor to generate photogenerated excitons or carriers, these photoexcited species need to migrate or diffuse to the reaction sites to drive the reaction. However, for excitons produced in organic semiconductors, the typically low exciton migration distances limit the participation of excitons in catalytic reactions. For carriers, the average free range of electrons or holes can make them collide and annihilate or complex during migration, limiting carriers' participation in catalytic reactions. These limitations are caused by the morphological characteristics of semiconductor catalysts. For the PTA nanosheets studied in this paper, photogenerated excitons and carriers are produced in excited molecules by light. However, typical water reduction reactions only occur at the edges of the nanosheets, which leads to surface area limitations on the apparent quantum efficiency of the actual hydrogen production.

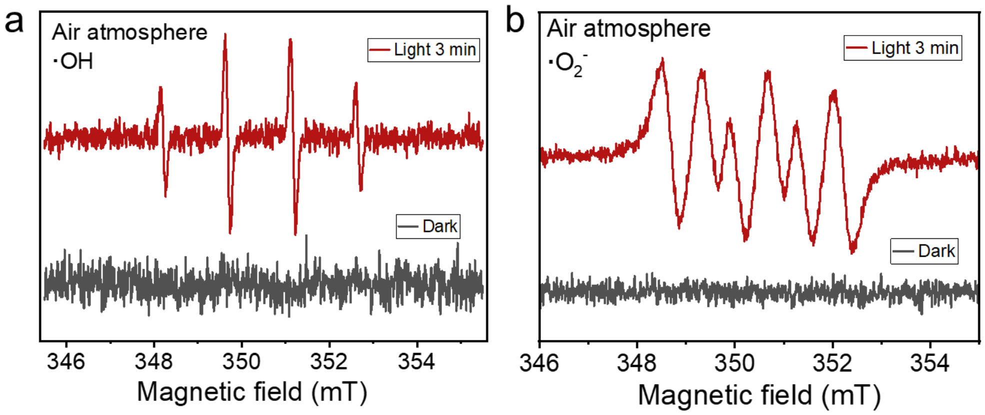

Supplementary Figure 51. A destination for carriers in PTCDA nanosheets. EPR test of the (a) hydroxyl radical (·OH) and (b) superoxide radical ($\cdot O_2^-$) signals captured by DMPO (0.1 M) in air atmosphere. It shows that ·OH and $\cdot O_2^-$ are produced by PTA nanosheets after light exposure in an air atmosphere.

Supplementary Note 5: Rationalization of second-order exponential functions to fit TAS kinetic processes

We have fitted the decay kinetics of photogenerated charge using a second-order exponential fit, where τ_1 indicates that the photogenerated carries are rapidly compounded after exciton dissociation and τ_2 indicates that the photogenerated carries are trapped during migration after dissociation.

In this study, the PTA nanosheets show an ordered crystal structure, which was confirmed by XRD as well as by electron diffraction patterns (Figure 1). According to the previous reports [12], excitons of highly crystalline organic materials are not prone to exciton-exciton annihilation because of the large effective volume, which is expected to give favourable exponential dynamics. From the results, we fit the TAS data according to the second-order exponential and all the R^2 can reach above 0.95, and the residual analysis shows that the mathematical model has a high confidence level.

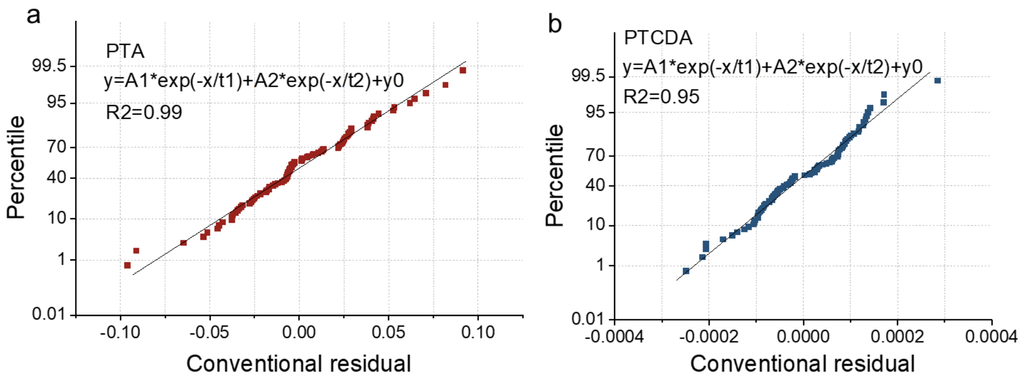

Supplementary Figure 52. Rationalization of fitting TAS dynamics using second-order exponential functions. Residual scores from second-order exponential fits to

TAS data for (a) PTA and (b) PTCDA.

Supplementary Note 6: Excluding the influence of morphology on the behavior of photogenerated charges

To exclude the effect of morphology on photogenerated charge, we modulated the synthesis of PTA (Supplementary Fig. 53a-c) and PTCDA (Supplementary Fig. 54a-c) with different morphologies.

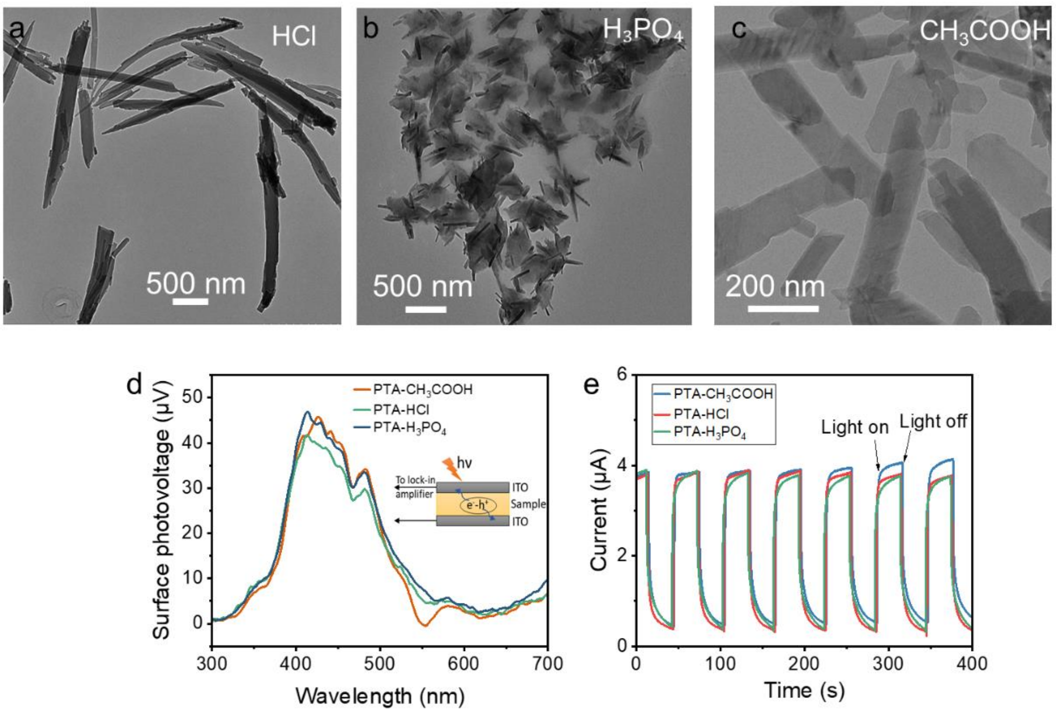

Supplementary Figure 53. The morphology of PTA on the behavior of photogenerated charges. Morphology of PTA regulated by (a) HCl, (b) H_3PO_4 and (c) CH_3COOH as acid. (d) Surface photovoltage and (e) current of PTA with different morphologies. Different morphologies were obtained by changing the type of acid (called solution B in the preparation method) used to prepare the PTA supramolecular. When HCl is used, the PTA formed is in strips of ~2.0 μm in length. When H_3PO_4 was

used, both short strips and sheets were available. The ~520 nm long PTA nanosheets shown in the main text were obtained using CH_3COOH.

The photogenerated charge properties are then explored for different morphologies of PTA. Surface photovoltage is a means of characterizing the accumulation of photogenerated charge on the surface. There are no significant differences in the SPV signals of the three morphologies of PTA (Supplementary Fig. 53d). In addition, their transient photocurrent signals are also relatively consistent (Supplementary Fig. 53e). This suggests that the effect of morphology on the photogenerated charge movement of PTA is minimal.

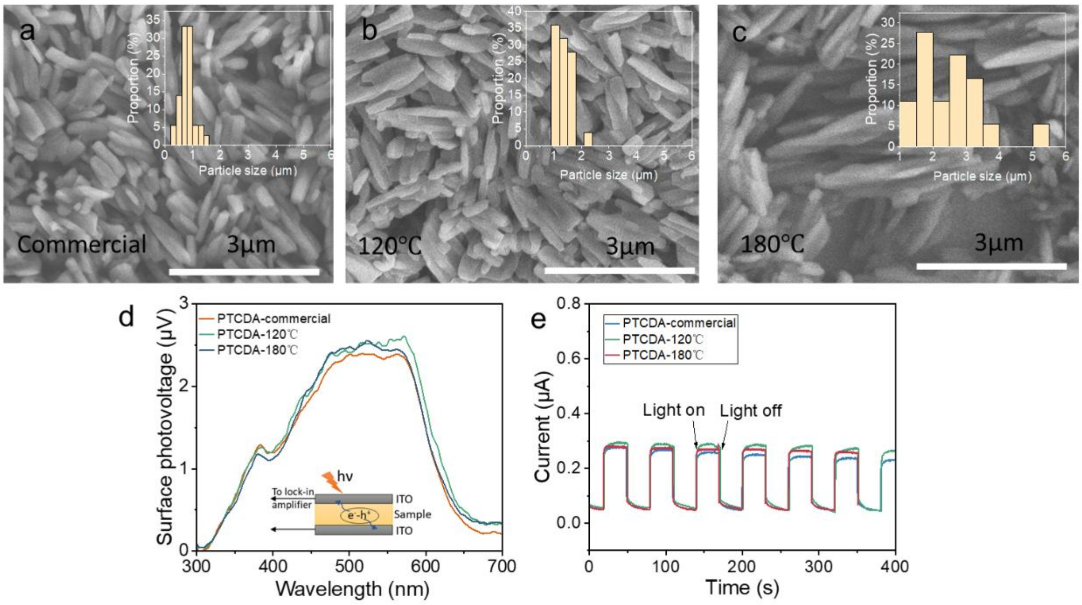

Supplementary Figure 54. The morphology of PTCDA on the behavior of photogenerated charges. SEM morphology of (a) commercial PTCDA, heat treatment at (b) 120°C and (c) 180°C. (d)Surface photovoltage and (e) current of PTCDA with different morphologies. The commercially available PTCDA was heated at 120°C and

180°C to obtain shapes with different lengths. The average rod lengths of commercially purchased, 120°C heat treated and 180°C heat treated PTCDA were 0.8 μm, 1.4 μm and 2.6 μm respectively.

To make the conclusions clearer, we also obtained PTCDA with different morphologies (Supplementary Fig. 54a-c) by heat treatment and characterized them in the same way as PTA. The PTCDA with different morphologies showed similar SPV (Supplementary Fig. 54d) and photocurrent (Supplementary Fig. 54e) signals to each other. This indicates that the effect of morphology on the photogenerated charge of PTCDA can also be neglected. Therefore, based on the above discussion, we conclude that the effect of morphology on the behavior of photogenerated charges can be ignored.

Supplementary Note 7: Factors affecting the morphology of PTA nanosheets

We have explored the effect of solution B and A concentration ratios, and stirring time on the morphology of PTA nanosheets. As described in Methods, KOH was used as the A solution and acetic acid as the B solution. When A: B was increased from 14:1 to 136:1, the resulting precipitates were still nanosheets, but the length of the nanosheets decreased from ~4.1 um to ~0.6 um. This was attributed to the ionic strength and polarity affecting the nucleation of the crystals' nucleation and the nanosheets' growth kinetics.

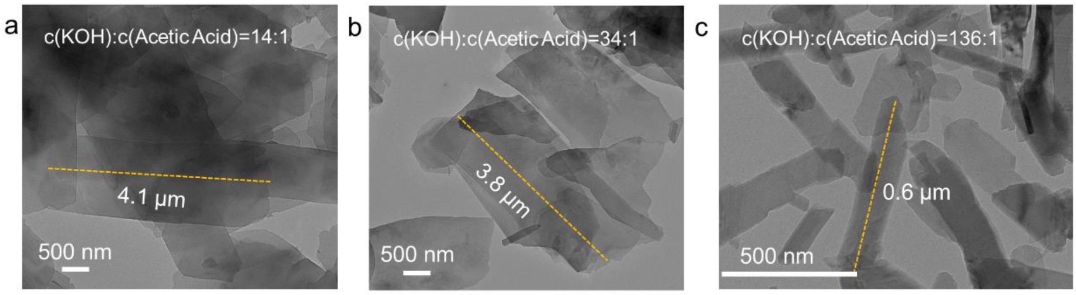

Supplementary Figure 55. Effect of concentration on the morphology of PTA. The morphology of PTA regulated under condition c (KOH): c (Acetic Acid) =14:1 (a), 34:1 (b) and 136:1 (c).

As the stirring time was extended from 10 min to 24 h, the precipitates were all two-dimensional nanosheets with no significant change in morphology. Attributed to the thermodynamic equilibrium reached, the nanosheets are stable thermodynamic morphologies.

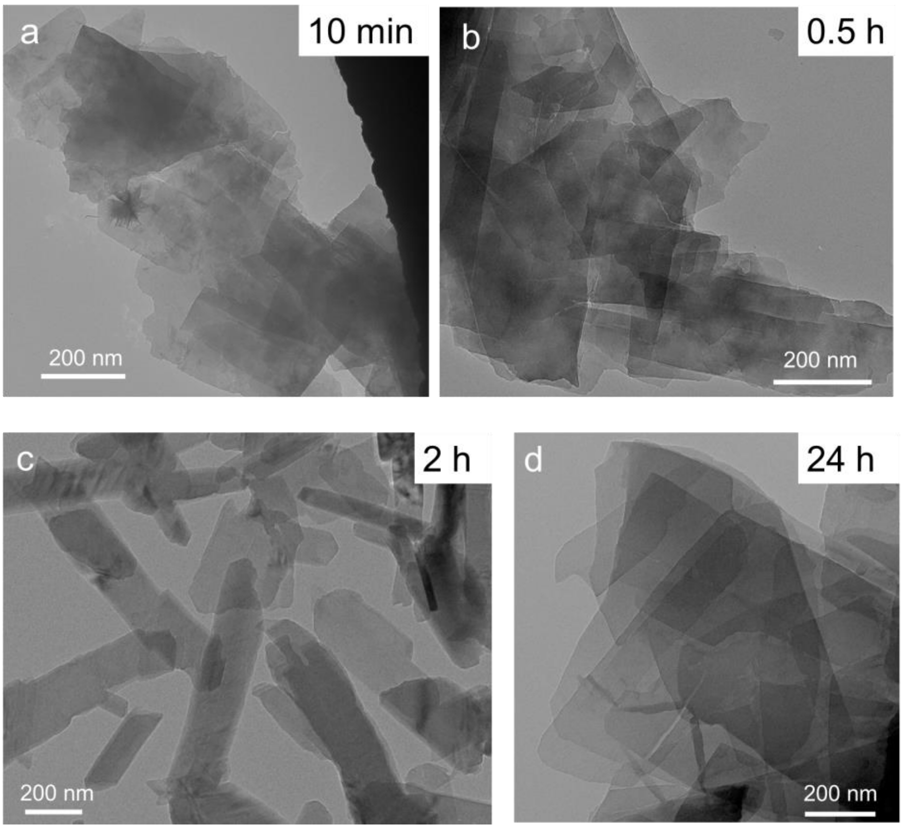

Supplementary Figure 56. Effect of reaction time on the morphology of PTA. The morphology of PTA prepared by stirring for (a) 10 min, (b) 0.5 h (c) 2 h and (d) 24 h.

Supplementary Note 8: Explain the stability of PTA nanosheets

We reveal the stability of PTA nanosheets in four ways. Firstly, the PTA molecules are assembled into long range and orderly PTA nanosheets by π-π stacking. Effective overlap occurs between the molecular orbitals of the perylene rings, resulting in stable aggregates. The photogenerated charges generated by PTA under light irradiation leave the domain through the π-orbitals of the interacting molecules, which makes PTA nanosheets less susceptible to damage.

Secondly, the reactive oxygen species produced by PTA nanosheets under light irradiation were characterized by EPR, as shown in the Supplementary Figure 57, where the photogenerated electrons of PTA are trapped by dissolved oxygen to form $\cdot O_2^-$ in an air environment. It further reacts with H^+ to form $\cdot OH$. However, the production of superoxide and hydroxyl radicals was barely detected in the nitrogen atmosphere, proving that the production of these reactive oxygen species is dependent on the oxygen atmosphere. In this work, the photocatalytic hydrogen production experiments of PTA nanosheets were carried out under vacuum conditions, so that the generation of reactive oxygen species was strongly inhibited, thus circumventing the interference of the reactive oxygen component with the photocatalysis itself.

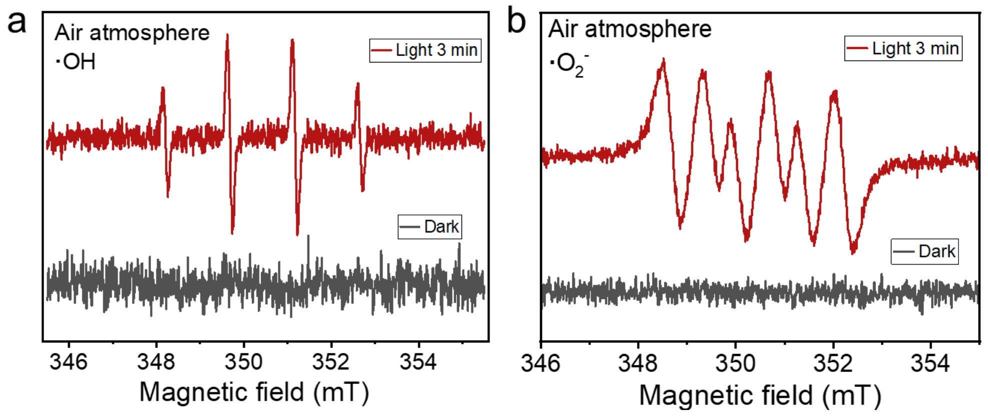

Supplementary Figure 57. The reactive oxygen species produced by PTA nanosheets under at atmosphere. EPR test of ·OH (a) and $\cdot O_2^-$ (b) production by PTA nanosheets after 3 minutes of light exposure.

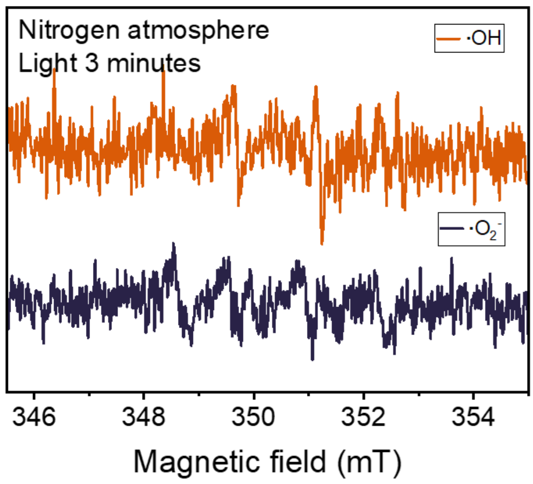

Supplementary Figure 58. The reactive oxygen species produced by PTA nanosheets at nitrogen atmosphere. EPR test of ·OH and $\cdot O_2^-$ production by PTA nanosheets after 3 minutes of light exposure.

Furthermore, even though traces of oxygen remaining in the hydrogen production environment led to the generation of small amounts of reactive oxygen species under

irradiation. The PTA molecule possesses the large conjugated structure of perylene, which are difficult for the reactive oxygen species to oxidize and decompose.

Finally, the presence of ascorbic acid in the photocatalytic hydrogen production half-reaction is highly oxidizable and the reactive oxygen species generated on the surface of the PTA nanosheets would preferentially react with the thermodynamically easy ascorbic acid.

In conclusion, the long-range ordered structure of PTA, and the hydrogen production environment that is not conducive to the generation of reactive oxygen species ensure the stability of PTA nanosheets.

3. Supplementary Methods

Supplementary Methods 1: Photocatalytic hydrogen production unit

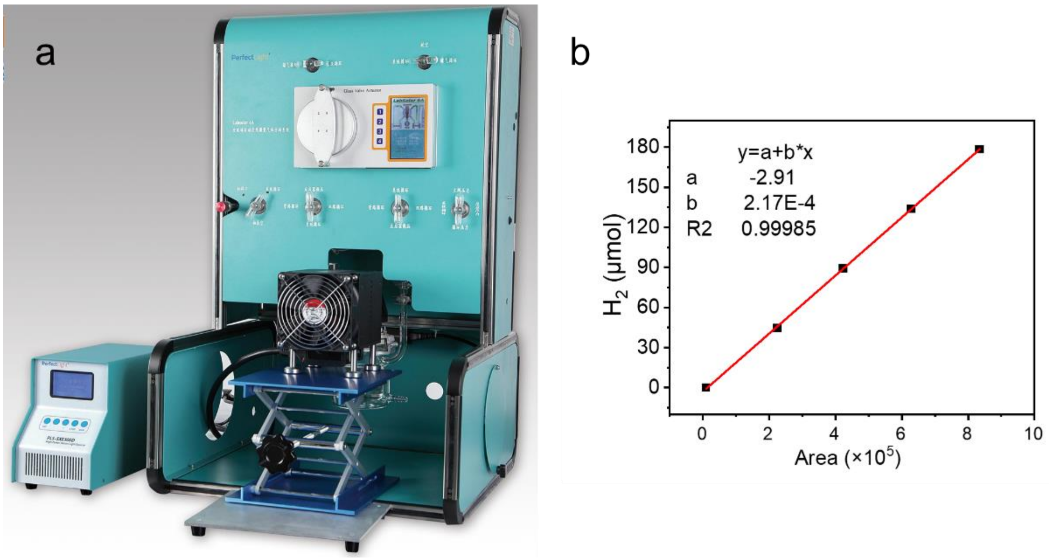

Supplementary Figure 59. Apparatus for testing photocatalytic HER performance. (a) Photograph of the photocatalytic on-line analytical system (Labsolar-6A, PerfectLight). (b) The calibration curves of photocatalytic H_2 evolution.

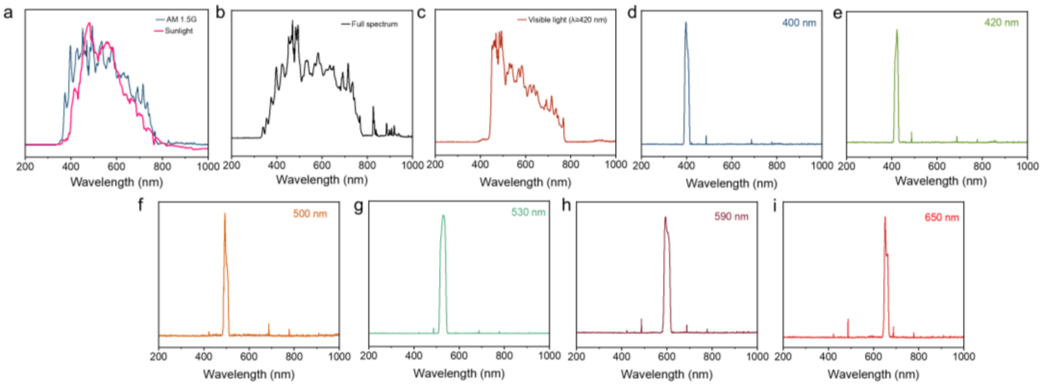

Supplementary Figure 60. The spectra of the different light sources. (a) AM 1.5G and sunlight, (b) full spectrum, (c) visible light, (d) 400 nm, (e) 420 nm, (f) 500 nm, (g) 530 nm, (h) 590 nm and (i) 650 nm.

Supplementary Methods 2: Determination of internal electric fields (IEF)

The Poisson equation describes the spatial distribution of the IEF, which shows that IEF is a function of surface potential and surface charge density [13-17].

$$F=\left(\frac{-2\rho V_s}{\varepsilon_0\varepsilon_r}\right)^{1/2} \qquad (8)$$

Where F is the internal electric fields magnitude, V_s is the surface potential, ρ is the surface charge density, Ɛ is the low-frequency dielectric constant, and ε_0 is the vacuum dielectric constant. The above equation reveals that the IEF magnitude is mainly determined by the surface voltage and the charge density because assuming other parameters are constant for perylene-based materials.

The surface potential V_s is obtained by Kelvin Probe Force Microscopy (KPFM) in the mode of surface potential.

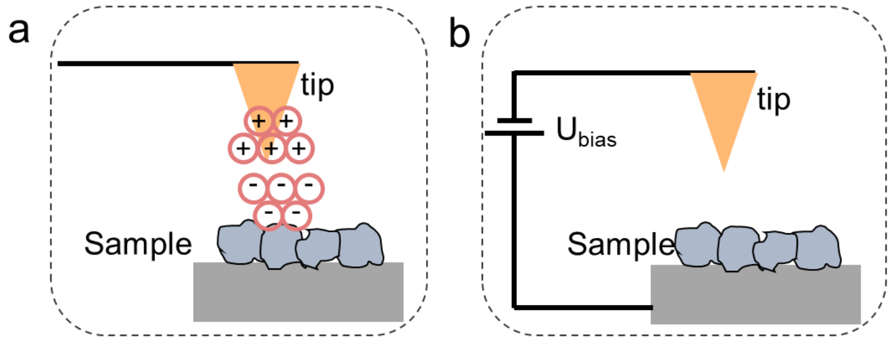

Supplementary Figure 61. Schematic diagram of Kelvin Probe Force Microscopy (KPFM) testing the surface potential of samples. The charge of the needle tip and sample surface of KPFM (a) before and (b) after the application of bias pressure.

Kelvin Probe Force Microscopy (KPFM) is a scanning technique for probing the

electrostatic potential distribution on the surface of a sample. Specifically, a known bias is applied between the sample and the tip of the needle (Supplementary Fig.61), and the surface potential distribution of the sample is then measured by KPFM.

The surface charge density ρ is tested by the zeta potential and then calculated using the model given by Gouy-Chapman[18]:

$$\sigma = \sqrt{8kT\varepsilon\varepsilon_0 n}\, sin\, h\left(\frac{ze_0\psi_0}{2kT}\right) \tag{9}$$

Among them, for the smaller Zeta potential value (ψ_0<50 mV), there are

approximately[19,20]:

$$\psi_0 = \zeta(1 + D/a_1)e^{\kappa D} \tag{10}$$

σ—surface charge density, C/m^2

k—the Boltzmann constant, $J \cdot K^{-1}$

T—absolute temperature, K

n—Number of electrolytes per unit volume, m^{-3}

e_0—electron charge, C

z—Electrolyte valence

ψ_0—Surface potential, V

a_1—Particle Stokes radius, m

ζ—zeta potential, it was measured at a concentration of 0.1 g/L samples, a NaCl concentration of 10 mmol/L and a temperature of 25 ° C.

κ^{-1}—Debye length, nm

D—Distance from sliding layer to particle surface

Therefore, we can simplify the IEF intensity as a function of the surface potential V_s and Zeta potential ζ. With the increases of V_s and ζ values, the strength of IEF increases, as shown below.

$$F=(AVs\sin h(\frac{Ze_0\zeta(1+\frac{D}{a_1})e^{\kappa D}}{2kT}))^{1/2} \tag{11}$$

Supplementary Methods 3: Surface photogenerated voltage (SPV) measurement mechanism.

The surface photovoltage (SPV) spectra are detected by a home-built apparatus, equipped with a lock-in amplifier (SR830, USA) synchronized with a xenon light source (CHF-XQ500W). The width of the slit is set to 3mm. The structure of the photovoltage sample cell is ITO glass - sample - ITO glass.

For N-type semiconductors, the Fermi level is higher than the surface state energy level. This leads to the migration of electrons from the native phase to the surface until the Fermi energy level reaches equilibrium, resulting in the accumulation of negative charge at the surface. Under light irradiation, photogenerated charge migrates to the surface. The SPV signal is the difference between the surface voltage in the light and the surface voltage in the dark. Therefore, the stronger the SPV signal, the better the separation of the photogenerated carriers.

4. Supplementary Tables

Supplementary Table 1. Grain size in dry and water state obtained by XRD

	Dry			Water			Change ratio
	FW (rad)	θ(degree)	D(Å)	FW (rad)	θ(degree)	D(Å)	
Sample	0.011	8.51	131.29	0.010	8.51	136.95	4%
	0.006	11.39	216.00	0.006	11.37	245.80	14%
External standard	0.003	14.14	440.29	0.003	14.15	554.91	26%
	0.004	23.57	393.16	0.003	23.57	435.07	11%
	0.004	27.98	363.09	0.004	27.98	402.52	11%

Supplementary Table 2. Summary of Pawley refinement results of PXRD

	System	a / Å	b / Å	c / Å	α /°	β /°	γ /°	V / Å3
PTA	Monoclinic	14.704	8.382	10.578	90	90	90	1303.73

The Pawley refined PXRD pattern with the *P212* space group gave the unit cell parameters of a =14.704 Å b = 8.382 Å, c = 10.578 Å, $\alpha = \beta = \gamma$ = 90°. The reproducible parameters are in line with the experimentally observed pattern with a negligible difference.

Supplementary Table 3. The summary of the photocatalytic performances of photocatalysts for hydrogen evolution from water splitting.

Photocatalyst	Light source (nm)	Sacrificial agent	HER (μmol h^{-1})	HER (mmol h^{-1} g^{-1})	Mass of Photocatalyst (mg)	Cocatalyst (Wt%)	Ref.
PTA	Full light	AA	826.28	118.04	7	4.64	This work
PTA	AM 1.5G	AA	292.6	41.8	7	4.64	This work
Poly(dibenzothiophene-S,S-dioxide) with K2HPO4	>420	TEOA	442	44.2	10	3w% Pt	21
Mesoporous TiO2	Full light	Methanol	--	3.57	--	3w% Au	22
NiS/Zn x Cd 1- x S	>420	Na2S and Na2SO3	839	16.78	50	NiS	23
CTF-BT/ Th-1	>420	TEOA	330	6.6	50	Pt (3 wt%)	24
CTF-1-100W	>420	TEOA	280	5.6	50	Pt (2.01 wt%)	25
TPPS/C-60	Full light	AA	276.55	34.57	8	6%	26
Py-HTP-BT-COF	>420	AA	177.50	8.875	20	5%	27
PTB7-Th:EH-IDTBR NP	350-800	AA	120	60	2	10% Pt	28
Phosphonate-Based MOFs	Full	TEOA	117.3	2.346	50	Pt	29
P10	>420	TEA	90	3.6	25	--	30
ZnTCPP	Full light	AA	87.175	3.487	25	3	31
L-PyBT	>420	TEOA	80	1.6	50	3w% Pt	32
PyTz-COF	AM 1.5G	AA	72.52	2.072	35	8	33
FS-TEG	>420	TEA	70	2.8	25	--	34
FS-COF	>420	AA	20	4	5	Pt (3 wt%)	35

Supplementary Table 4. The intensity of light used in AQE tests

Wavelength (nm)	350	400	420	450	475	500	530	590
Intensity (mW cm^{-2})	40	52	40	45	40	61	63	60

5. Supplementary References

1. Qureshi, M. & Takanabe, K. Insights on Measuring and Reporting Heterogeneous Photocatalysis: Efficiency Definitions and Setup Examples. *Chemistry of Materials* **29**, 158-167, (2017).

2. Rahman, M., Tian, H. & Edvinsson, T. Revisiting the Limiting Factors for Overall Water-Splitting on Organic Photocatalysts. *Angew. Chem. Int. Ed.* **59**, 16278-16293, (2020).

3. Kisch, H. & Bahnemann, D. Best Practice in Photocatalysis: Comparing Rates or Apparent Quantum Yields? *J Phys Chem Lett* **6**, 1907-1910, (2015).

4. Vdović, S. *et al.* Excited state dynamics of β-carotene studied by means of transient absorption spectroscopy and multivariate curve resolution alternating least-squares analysis. *Phys. Chem. Chem. Phys.* **15**, 20026-20036, (2013).

5. Chen, X. J., Wang, J., Chai, Y. Q., Zhang, Z. J. & Zhu, Y. F. Efficient Photocatalytic Overall Water Splitting Induced by the Giant Internal Electric Field of a g-C3N4/rGO/PDIP Z-Scheme Heterojunction. *Adv. Mater.* **33**, 7, (2021).

6. Wolff, C. M. *et al.* All-in-one visible-light-driven water splitting by combining nanoparticulate and molecular co-catalysts on CdS nanorods. *Nature Energy* **3**, 862-869, (2018).

7. Williams, A. T. R., Winfield, S. A. & Miller, J. N. Relative fluorescence quantum yields using a computer-controlled luminescence spectrometer. *Analyst* **108**, 1067-1071, (1983).

8. Kraner, S., Scholz, R., Plasser, F., Koerner, C. & Leo, K. Exciton size and binding energy limitations in one-dimensional organic materials. *JOURNAL OF CHEMICAL PHYSICS* **143**, (2015).

9. Kraner, S., Prampolini, G. & Cuniberti, G. Exciton Binding Energy in Molecular Triads. *JOURNAL OF PHYSICAL CHEMISTRY C* **121**, 17088-17095, (2017).

10. An, Z. S. *et al.* High electron mobility in room-temperature discotic liquid-crystalline perylene diimides. *ADVANCED MATERIALS* **17**, 2580, (2005).

11. Xue, D. A Simple Method for Determing Dielectric Constants of Materials with the Similar Crystal Structure. *Chem. Res. Chin. Univ.* **11**, 4, (2000).

12. Evans, A. M. *et al.* Seeded growth of single-crystal two-dimensional covalent organic frameworks. *Science* **361**, 52-57, (2018).

13. Li, J., Zhang, L., Li, Y. & Yu, Y. Synthesis and internal electric field dependent photoreactivity of Bi3O4Cl single-crystalline nanosheets with high {001} facet exposure percentages. *Nanoscale* **6**, 167-171, (2014).

14. Li, J., Zhan, G., Yu, Y. & Zhang, L. Superior visible light hydrogen evolution of Janus bilayer junctions via atomic-level charge flow steering. *Nat. Commun.* **7**, 11480, (2016).

15. Pierre Lefebvre, J. A. g., Bernard Gil, and Henry Mathieu. Time-resolved photoluminescence as a probe of internal electric fields in GaN-(GaAl)N quantum wells. *Phys. Rev.*

B **59**, 5, (1999).

16. Jin Seo Im, H. K., J. Off, A. Sohmer, F. Scholz, and A. Hangleiter. Reduction of oscillator strength due to piezoelectric fields in GaN/AlxGa1-xN quantum wells. *Phys. Rev. B* **57**, 3, (1997).

17. Morello, G. *et al.* Intrinsic optical nonlinearity in colloidal seeded grown CdSe/CdS nanostructures: Photoinduced screening of the internal electric field. *Phys. Rev. B* **78**, 8, (2008).

18. Shaw, D. J. *Introduction to colloid and surface chemistry*. 170-180 (London ; Butterworths, FMT:BK.3rd ed., 1980).

19. Shaw, D. *Introduction to colloid and surface chemistry* 170-180 (Butterworth-Heinemann, 1992).

20. C. J. VAN OSS, R. F. G., AND P. M. COSTANZO. DLVO and non-DLVO interactions in hectorite. *Clays and Clay Minerals* **38**, 151-159, (1990).

21. Shu, G., Li, Y., Wang, Z., Jiang, J.-X. & Wang, F. Poly(dibenzothiophene-S,S-dioxide) with visible light-induced hydrogen evolution rate up to 44.2 mmol h−1 g−1 promoted by K2HPO4. *Applied Catalysis B: Environmental* **261**, 118230, (2020).

22. Xiong, H. *et al.* Controllable Synthesis of Mesoporous TiO2 Polymorphs with Tunable Crystal Structure for Enhanced Photocatalytic H2 Production. *Advanced Energy Materials* **9**, 1901634, (2019).

23. Zhao, X. *et al.* An Efficient, Visible-Light-Driven, Hydrogen Evolution Catalyst NiS/ZnxCd1−xS Nanocrystal Derived from a Metal–Organic Framework. *Angew. Chem. Int. Ed.* **57**, 9790-9794, (2018).

24. Huang, W., He, Q., Hu, Y. & Li, Y. Molecular Heterostructures of Covalent Triazine Frameworks for Enhanced Photocatalytic Hydrogen Production. *Angew. Chem. Int. Ed.* **58**, 8676-8680, (2019).

25. Xie, J. *et al.* Efficient visible light-driven water oxidation and proton reduction by an ordered covalent triazine-based framework. *Energy Environ. Sci.* **11**, 1617-1624, (2018).

26. Yang, J., Jing, J. F. & Zhu, Y. F. A Full-Spectrum Porphyrin-Fullerene D-A Supramolecular Photocatalyst with Giant Built-In Electric Field for Efficient Hydrogen Production. *Advanced Materials*.

27. Chen, W. *et al.* Modulating Benzothiadiazole-Based Covalent Organic Frameworks via Halogenation for Enhanced Photocatalytic Water Splitting. *Angew. Chem. Int. Ed.* **59**, 16902-16909, (2020).

28. Kosco, J. *et al.* Enhanced photocatalytic hydrogen evolution from organic semiconductor heterojunction nanoparticles. *Nat. Mater.* **19**, 559-565, (2020).

29. Zhu, Y.-P. *et al.* Highly Stable Phosphonate-Based MOFs with Engineered Bandgaps for Efficient Photocatalytic Hydrogen Production. *Adv. Mater.* **32**, 1906368, (2020).

30. Sachs, M. *et al.* Understanding structure-activity relationships in linear polymer

photocatalysts for hydrogen evolution. *Nat. Commun.* **9**, 4968, (2018).

31. Jing, J., Yang, J., Zhang, Z. & Zhu, Y. Supramolecular Zinc Porphyrin Photocatalyst with Strong Reduction Ability and Robust Built-In Electric Field for Highly Efficient Hydrogen Production. *Adv. Energy Mater.* **11**, 2101392, (2021).

32. Cheng, C. *et al.* The effect of molecular structure and fluorination on the properties of pyrene-benzothiadiazole-based conjugated polymers for visible-light-driven hydrogen evolution. *Polymer Chemistry* **9**, 4468-4475, (2018).

33. Li, W. *et al.* Thiazolo[5,4-d]thiazole-Based Donor–Acceptor Covalent Organic Framework for Sunlight-Driven Hydrogen Evolution. *Angew. Chem. Int. Ed.* **60**, 1869-1874, (2021).

34. Woods, D. J. *et al.* Side-chain tuning in conjugated polymer photocatalysts for improved hydrogen production from water. *Energy Environ. Sci.* **13**, 1843-1855, (2020).

35. Wang, X. *et al.* Sulfone-containing covalent organic frameworks for photocatalytic hydrogen evolution from water. *Nature Chemistry* **10**, 1180-1189, (2018).
